# Secreted sphingomyelins modulate low mammary cancer incidence observed in certain mammals

**DOI:** 10.1038/s41598-020-77639-1

**Published:** 2020-11-25

**Authors:** Melissa M. Ledet, Rebecca M. Harman, Jennifer C. Fan, Emily Schmitt-Matzen, Maria Elena Diaz-Rubio, Sheng Zhang, Gerlinde R. Van de Walle

**Affiliations:** 1 Baker Institute for Animal Health, College of Veterinary Medicine, Cornell University, 235 Hungerford Hill Road, Ithaca, NY 14853 USA; 2 Proteomic and Metabolomics Facility, Cornell University, Ithaca, NY 14853 USA

**Keywords:** Cell biology, Breast cancer, Cancer models

## Abstract

Determining mechanisms that naturally protect species from developing cancer is critical in order to prevent and treat cancer. Here, we describe a novel cancer-suppressing mechanism, via the secretion of bioactive factors by mammary cells, that is present in domesticated mammals with a low mammary cancer incidence. Specifically, these bioactive factors induced triple-negative breast cancer cell (TNBC) death in vitro and reduced tumorigenicity in a xenograft TNBC mouse model in vivo. RNA deep sequencing showed significant downregulation of genes associated with breast cancer progression in secretome-cultured TNBC cells. Further in-depth multi-omics analysis identified sphingomyelins as key secreted factors, and their role was confirmed via inhibition of the sphingomyelin signaling pathway. We speculate that secreted sphingomyelins in the mammary gland of mammals with a naturally low incidence of mammary cancer mediate the elimination of cancer cells. This study contributes to the growing list of protective mechanisms identified in cancer-proof species.

## Introduction

It is increasingly recognized that much can be learned about cancer incidence from comparative cross-species studies. Indeed, several sophisticated and effective cancer suppression mechanisms have been discovered in primarily long-lived wild mammals that are naturally resistant to this disease^1,2^. For example, it has been found that naked mole rats, the longest-living rodent species, avoid cancer via (i) the production of large quantities of high molecular mass hyaluronan, (ii) a relatively stable epigenome, (iii) higher copy numbers of genome maintenance genes, and (iv) apoptosis of cells that have lost a tumor suppressor gene^3–6^. Elephants are another long-lived wild mammal with a very low cancer mortality rate. Studies in this species have identified functional duplicates of the master tumor suppressor gene *TP53*, as well as a re-functionalized *LIF6* pseudogene, both of which are associated with an enhanced p53-dependent DNA damage response. This response results in the clearing of damaged cells via apoptosis as opposed to the repair of flawed cells, which may lead to oncogenic mutations^7,8^.

Interestingly, variable cancer dynamics across species are not only observed in long-lived wild mammals, but are seen in domesticated animals as well. An important observation in the breast cancer field is that mammary cancer occurs in many domesticated animals, but varies drastically in incidence rate. For example, un-spayed dogs and cats are diagnosed with mammary cancer at a frequency of up to 26%, which is near double the incidence rates observed in women, but mammary cancer is reported to only affect 0.03 to 2% of horses and is rarely diagnosed in pigs and ruminants, despite the development of other types of cancers in these animals^9,10^. Although explanations for variations in mammary cancer incidence, such as differences in diet, environmental exposures, or circulating hormone levels, have been put forward over the years, a univocal explanation for this observation remains elusive. As studying cancer-resistant wild mammals has led to a better understanding of novel anti-cancer mechanisms, work on mammals with a low incidence of mammary cancer can provide invaluable insights into the mechanisms underlying resistance to this particular type of cancer as well. We recently reported on a mechanism by which mammosphere-derived epithelial cells (MDEC) from horses, a species with a low mammary cancer incidence, respond to DNA damage by preferentially undergoing apoptosis as opposed to repair^11^. Interestingly, this mechanism shows compelling similarities with the enhanced apoptosis in response to DNA damage observed in fibroblasts from elephants^7^, and thus, may serve as a protection action that is evolutionarily conserved across species.

In addition to initiating pro-apoptotic functions in response to DNA damage, mammals with low mammary cancer incidence may also protect themselves by secreting bioactive factors with anti-cancer properties in order to eliminate cancer cells when they arise. This concept is illustrated in a study in which human uterine cervical cells were shown to secrete bioactive factors with anti-tumoral potential^12^. Such cell-secreted bioactive factors, collectively termed the secretome, include macromolecules such as proteins/peptides, DNA, RNA, and small molecules such as glycans, lipids, and metabolites. The secretome plays an essential role in a wide variety of physiological, such as regeneration, and pathophysiological, such as tumor growth and metastasis, processes^13,14^. Importantly, the secretome is proposed to be of broad medical relevance due to the presence of cell-secreted proteins and other components that can be developed into pharmacologically active drug compounds^15^.

To explore the potential of cell-secreted products to protect against mammary cancer, we collected the secretome from MDEC isolated from various domesticated mammals, both with low and high mammary cancer incidence, and evaluated effects of the secretome on human breast cancer cells in vitro and in vivo. Our novel findings were that the MDEC secretome from mammals with a low mammary cancer incidence, such as equines and bovines, induced triple-negative breast cancer (TNBC) cell death in vitro and reduced tumorigenicity of these cells in vivo. Sphingomyelins were identified as key secreted factors, and their functional role was confirmed via inhibition of the sphingomyelin signaling pathway. Collectively, this study contributes to the growing list of protective mechanisms identified in species with no or low cancer incidence, and could provide the rationale for the development of novel therapies for breast cancer treatment and prevention.

## Results

### The secretome from equine mammosphere-derived epithelial cell (EqMDEC) specifically affects the viability of human triple negative breast cancer (TNBC) cell lines in vitro and downregulates genes that are associated with breast cancer progression

**Figure 1:**
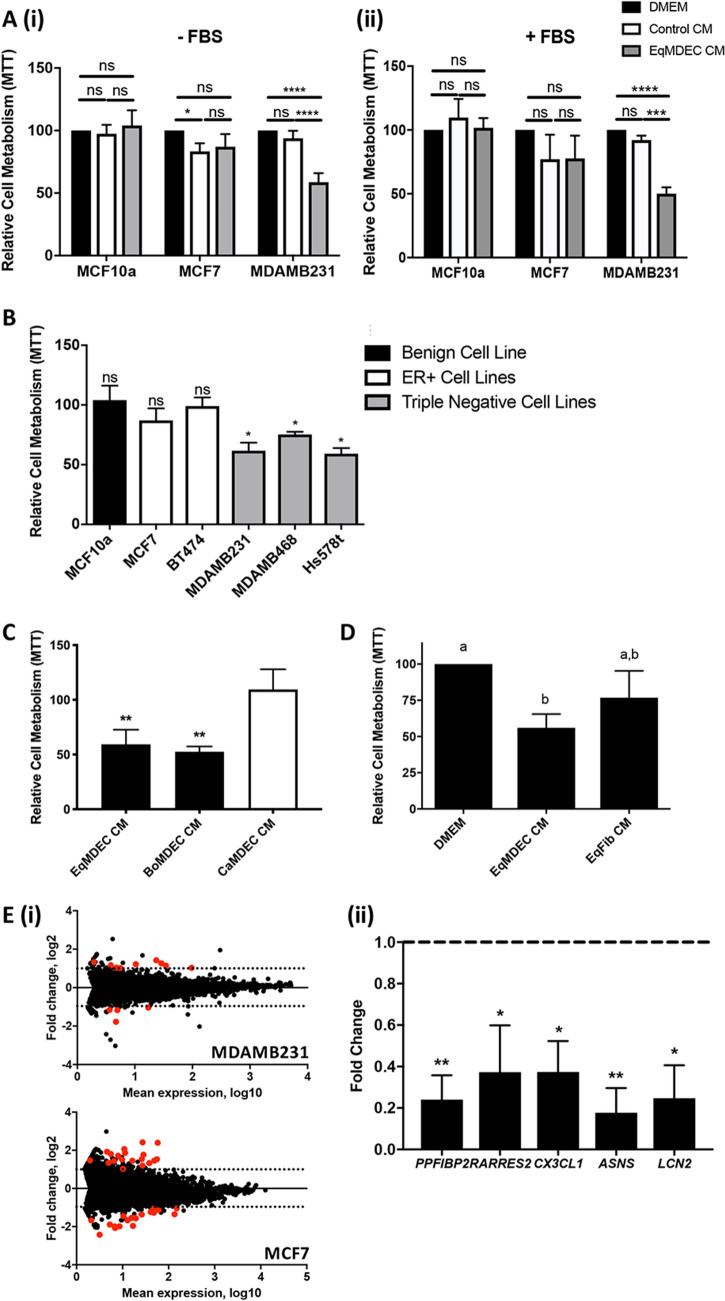
The secretome of EqMDEC specifically reduces viability of TNBC cells in vitro. MTT assays of human cell lines cultured for 48 h in the presence of conditioned medium (CM) or medium control from EqMDEC. Values are expressed relative to DMEM. (**A**) MCF10a, MCF-7, and MDA-MB-231, cultured with EqMDEC CM, self-CM (Control CM), or DMEM, in the absence (i) or presence (ii) of 10% fetal bovine serum (FBS). (**B**) MCF10a, estrogen receptor-positive (ER^+^) breast cancer cell lines, and TNBC cell lines, cultured with EqMDEC CM or DMEM. MTT assays of MDA-MB-231 cultured with EqMDEC CM, canine (Ca) MDEC-CM, bovine (Bo) MDEC-CM, or DMEM (**C**); and with EqMDEC CM or CM from equine mammary fibroblasts (EqFib) (**D**). (**E**) MA analysis showing differentially expressed genes (DEG) in MDA-MB-231 and MCF-7 that were either cultured in control conditions (own CM) or in EqMDEC CM for 12 h (p < 10^−6^). DEG are highlighted in red (i) and qRT-PCR of 5 genes detected by RNA sequencing to be differentially expressed in EqMDEC CM-cultured MDA-MB-231 cells (ii). Significant differences are either depicted by asterisks: *p < 0.05, **p < 0.01, ***p < 0.001, ****p < 0.0001, or by different letters. ns: not significant, n = 3. Data are presented as the mean ± standard deviation.

To explore the potential anti-cancer effects of the secretome from MDEC from domesticated mammals with a low cancer incidence, the normal human breast epithelial cell line MCF10a, the estrogen receptor-positive (ER^+^) breast cancer cell line MCF-7, and the triple-negative breast cancer (TNBC) cell line MDA-MB-231 were incubated with conditioned medium (CM) from equine MDEC (eqMDEC) and changes in cell metabolism, as a proxy for cell viability, were assessed using MTT assays. No effect on MCF10a and MCF-7 viability was detected upon exposure to eqMDEC CM when compared to medium alone (DMEM) or self-CM (control CM), with exception of a slight, but significant, decrease in viability of MCF-7 cells treated with control CM (Fig. 1A(i)). In contrast, a significant decrease in MDA-MB-231 viability was found when this cell line was cultured with eqMDEC CM (Fig. 1A(i)), which was dose-dependent with a cut-off at a 1:3 dilution of eqMDEC CM (Supplementary Fig. 1A). To ensure that observed effects were due to a factor secreted by eqMDEC and not just merely a consequence of using CM that may be depleted of nutrients, experiments were repeated in serum-rich media. Similar results were obtained, with the exception that control CM no longer decreased MCF-7 viability (Fig. 1A(ii)). To verify that cell death and not merely metabolic differences was assessed, experiments were repeated with additional measures of cytotoxicity, including lactate dehydrogenase (LDH) release assays and detection of caspase 3-positive cells. This corroborated our findings that the EqMDEC secretome initiates MDA-MB-231 cell death (Supplementary Fig. 1B). When additional ER^+^ and TNBC cell lines were included to determine whether the EqMDEC CM effects were specific for MCF-7 and MDA-MB-231 or more reflective of the general receptor status of breast cancer cells, we noticed a selective TNBC cell death (Fig. 1B). Next, we evaluated the viability of MDA-MB-231 incubated with MDEC CM from additional domesticated mammals, to determine whether the observed effect was equine-specific or more reflective of the species’ mammary cancer incidence status. We found that MDEC CM from bovines, a species with low mammary cancer incidence, but not from canines, a species with high mammary cancer incidence, also significantly reduced MDA-MB-231 viability (Fig. 1C). Finally, we evaluated the viability of MDA-MB-231 incubated with CM from tissue-matched equine mammary fibroblast cells (EqFib CM) and found that only EqMDEC, but not EqFib CM, induced significant MDA-MB-231 cell death (Fig. 1D), indicating that the observed effect is epithelial cell-specific. Collectively, these data show that the secretome of MDEC from domesticated mammals with a low, but not from those with a high mammary cancer incidence, causes significant cell death of TNBC cell lines in vitro, but does not affect normal mammary epithelial cells or ER^+^ breast cancer cell lines.

Using RNA deep sequencing, we next investigated transcriptional changes in MDA-MB-231 exposed to EqMDEC to start unraveling the underlying molecular mechanisms resulting in cell death in these target cells. Samples of MDA-MB-231 cultured with self-CM were included to control for any CM-related transcriptional changes, as well as samples of MCF-7 cultured with EqMDEC since no effect on viability was observed in these cells (Fig. 1A,B). Thirteen genes, with 9 up- and 4 downregulated, and 39 genes, with 20 up- and 19 downregulated, were identified as differentially expressed genes (DEG) in MDA-MB-231 and MCF-7, respectively, when cultured in EqMDEC CM compared to self-CM (Fig. 1E, Table 1). One gene, *TXNIP*, was upregulated in both cell lines, and was thus excluded from further analysis (Table 1). The remaining 12 DEG specific for EqMDEC CM-cultured MDA-MB-231 were further evaluated by qRT-PCR. Only 5 genes were confirmed to be significantly differentially expressed both by RNA deep sequencing and qRT-PCR and all were downregulated, namely *PPFIBP2*, *RARRES2*, *CX3CL1*, *ASNS*, and *LCN2* (Fig. 1E).

**Table 1:** Differentially expressed genes in MDA-MB-231 and MCF-7 cells after treatment with EqMDEC-CM, as identified by RNA sequencing.

MDA-MB-231				MCF-7			
Upregulated		Downregulated		Upregulated		**Downregulated**	
Gene	Fold change	Gene	Fold change	Gene	Fold change	*Gene*	*Fold Change*
*ADAM19*	0.488364	*ASB2*	2.328582	*ABCC3*	0.367341	*AHNAK*	2.292338
*COL6A3*	0.447725	ASNS*	2.402235	*ACOX2*	0.186668	*AMOTL1*	2.70862
*MYH16*	0.44397	***CX3CL1***	2.051974	*ADORA1*	0.237824	*CALCOCO1*	3.279585
*P2RY6*	0.291689	*GGT5*	2.027984	*AGPAT2*	0.447876	*CLMN*	2.771473
		***LCN2***	2.042948	*BMP7*	0.3148	*ELOVL2*	3.795495
		***PPFIBP2***	2.507134	*CCDC86*	0.424847	*FREM2*	2.033828
		***RARRES2***	2.204093	*DUSP5*	0.270298	*METTL7A*	2.735308
		*TXNIP*	2.677222	*ETV4*	0.253306	*MYOF*	2.743046
		*VIPR1*	2.218237	*ETV5*	0.31451	*NPY1R*	3.406242
				*FABP5*	0.391751	*OPTN*	3.497602
				*KRT80*	0.345063	*PDCD4*	2.510132
				*LAD1*	0.478078	*PGR*	2.54223
				*N4BP3*	0.415255	*RTN2*	4.185677
				*PRSS23*	0.342333	*SCIN*	2.895793
				*RET*	0.389265	*SELENBP1*	2.881265
				*SDC1*	0.339147	*SLC26A2*	3.631624
				*TGM2*	0.251892	*TP53INP1*	2.867714
				*TOMM40*	0.486226	*TXNIP*	5.277761
				*TUBB3*	0.457793	*YPEL3*	5.348536
				*VGF*	0.255917		

*Bolded genes: genes that were confirmed to be differentially expressed in MDA-MB-231 by qRT-PCR as well.

### The secretome from EqMDEC reduces xenograft tumor growth of MDA-MB-231 in vivo

**Figure 2:**
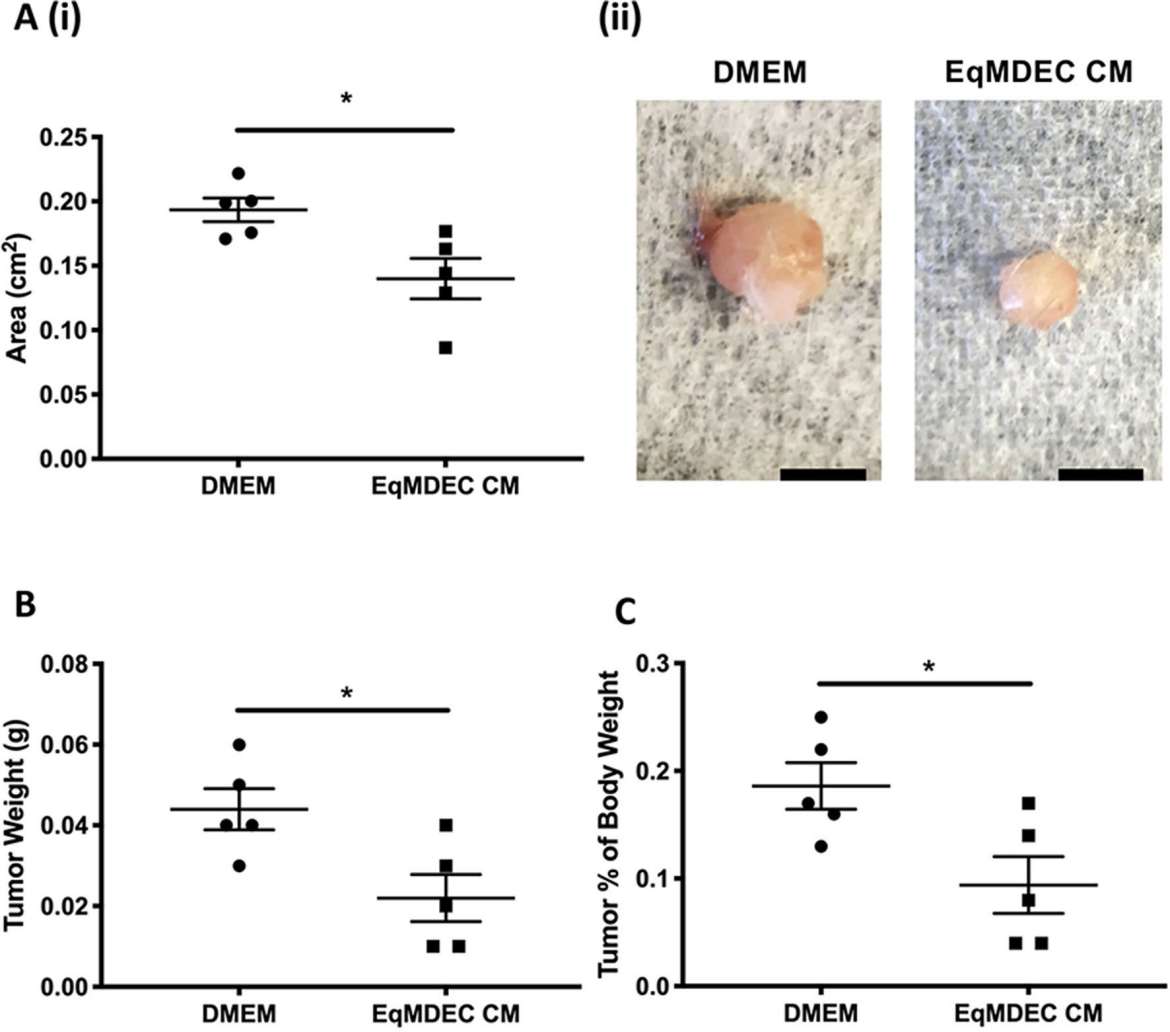
The secretome of EqMDEC reduces tumorigenicity in vivo. NSG mice were injected orthotopically with 3 × 10^6^ MDA-MB-231 cells in Matrigel. Two weeks later, mice were intratumorally injected with DMEM (control) or EqMDEC conditioned medium (CM) daily for two weeks. (**A**) Quantification of tumor area (**i**) and representative images of tumors removed from mice treated with either DMEM (control) or EqMDEC CM (**ii**). (**B**) Quantification of tumor weight. (**C**) Tumor weight expressed as a percentage of body weight. *p < 0.05, n = 5/group. Data are presented as the mean ± standard error.

Based on these solid in vitro data, we evaluated the effects of EqMDEC CM in vivo using a mouse xenograft model of TNBC. Tumors in the EqMDEC CM-treated group were visually smaller and had a significantly decreased area compared to the tumors in the DMEM-treated control group (Fig. 2A). Moreover, both overall tumor weight and tumor weight as a percentage of body weight, were significantly lower in the EqMDEC CM-treated group (Fig. 2B,C), demonstrating that the anti-cancer effects of the EqMDEC secretome function in vivo as well.

### The bioactive factor(s) in EqMDEC CM are small in size and temperature-stable

**Figure 3:**
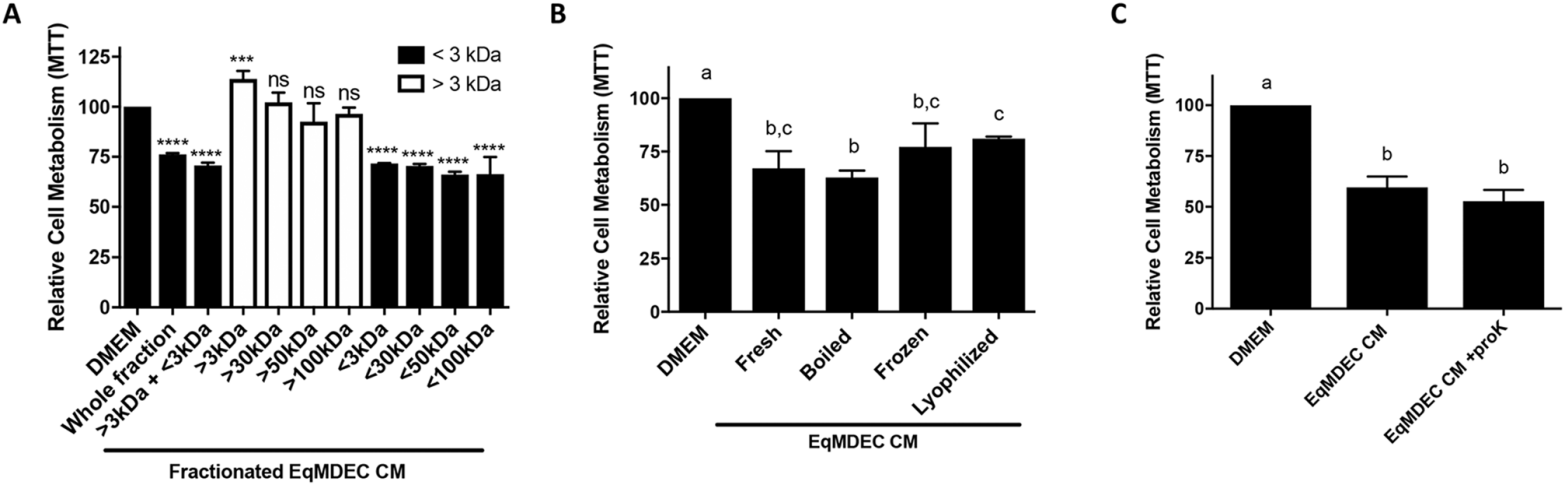
The bioactive factor(s) in EqMDEC CM are small in size and temperature-stable. MTT assays of MDA-MB-231 cultured for 48 h in the presence of EqMDEC CM that was manipulated, as described. DMEM controls were also included for each experiment. (**A**) EqMDEC CM was fractionated with centrifugation filters. (**B**) EqMDEC CM was either boiled at 100 °C for 10 min, frozen at − 80 °C for one week, or lyophilized and reconstituted to its original volume. (**C**) EqMDEC CM was pre-treated with proteinase K. Significant differences are either depicted by asterisks: ****p < 0.0001, or by different letters. ns: not significant, n = 3. Data are presented as the mean ± standard deviation.

To narrow down the nature of the EqMDEC secreted bioactive factor(s) with anti-cancer effects, we first fractionated the EqMDEC CM using centrifugal filters to determine the size range of the bioactive factor(s). All fractionated CM samples containing factors < 3 kDa significantly reduced MDA-MB-231 viability, whereas the fractions exclusive of factors < 3 kDa did not show any effect (Fig. 3A). In order to ensure the bioactive factors were not lost during the fractionation protocol, the > 3 kDa and < 3 kDA fractions were pooled together, which led to the same effects as unfractionated CM (Fig. 3A). Next, we subjected the EqMDEC CM to (i) boiling, (ii) freezing, and (iii) lyophilizing, all with reconstitution to original volume, and found that none of these pre-treatments altered the efficacy when compared to fresh (untreated) CM (Fig. 3B). Finally, EqMDEC CM was pre-treated with proteinase K in order to degrade proteins, which was confirmed using Coomassie blue staining (data not shown). Again, no difference in CM efficacy was observed (Fig. 3C). Experiments consisting of CM pre-treatments with RNase and lipid eliminators, to evaluate whether the bioactive factors could be miRNAs or lipids, respectively, were inconclusive, due to inherent cytotoxic effects on MDA-MB-231 (data not shown). Collectively, these results demonstrate that the bioactive factor(s) in the eqMDEC secretome are small compounds, most likely non-protein in nature, that can withstand temperature extremes and freeze-drying.

### In-depth “omics” analysis identified sphingomyelins in the equine MDEC secretome that contribute to reduced TNBC viability

**Figure 4:**
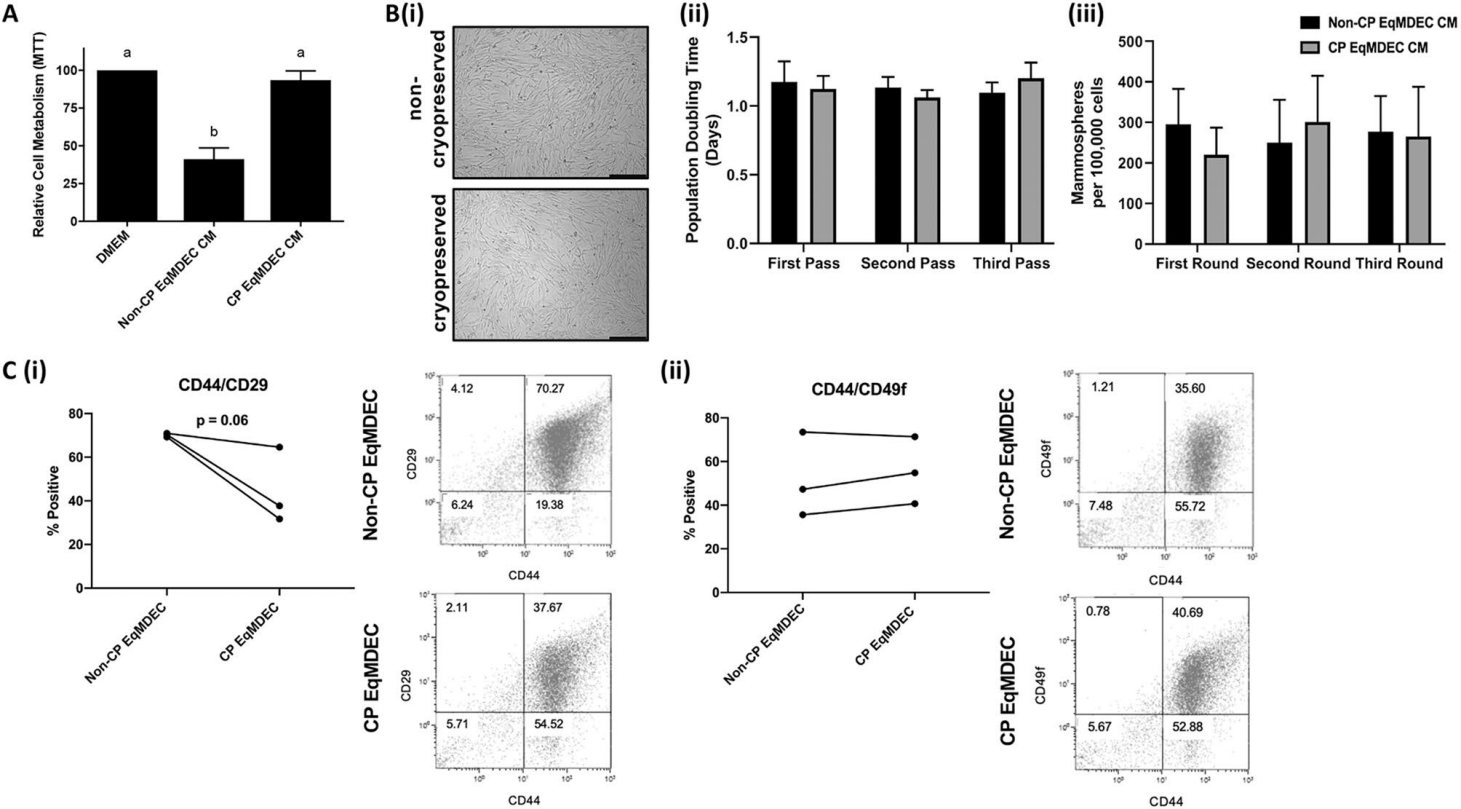
Cryopreservation of EqMDEC affects the bioactivity of the secretome. (**A**) MTT assays of MDA-MB-231 cells cultured for 48 h in the presence of CM that was collected either from freshly isolated EqMDEC (non-CP) or previously cryopreserved EqMDEC (CP). (**B**) Non-CP and CP Eq MDEC look similar morphologically (i), have similar population doubling times (PDT) (ii), and display similar mammosphere-forming capacities (iii). (**C**) Non-CP and CP Eq MDEC differ in the percentage of CD44/CD29 double-positive cells detected by flow cytometry (i) while the percent of CD44/CD49f. double-positive cells is similar regardless of cryopreservation status (ii). Significant differences are depicted by different letters, n = 3. Data are presented as the mean ± standard deviation.

To identify putative bioactive factors in the EqMDEC secretome, we took advantage of an interesting observation made during our experiments. We found that CM collected from EqMDEC that had been cryopreserved no longer affected the viability of MDA-MB-231, whereas CM collected from the same EqMDEC cultures that were freshly isolated significantly reduced MDA-MB-231 viability (Fig. 4A). This striking difference in CM efficacy provided us with a unique opportunity to identify the bioactive factor(s) with anti-cancer effects in the secretome of EqMDEC by directly comparing analytes present in CM from the same EqMDEC cultures that were either cryopreserved (CP) or fresh (non-CP). Cryopreservation of EqMDEC cultures did not change their morphology, population doubling time, and capacity to form mammospheres, when compared to non-cryopreserved cells (Fig. 4B). However, flow cytometry analysis revealed a reduction in a subpopulation of CD44/CD29, but not CD44/CD49f, -double positive cells upon cryopreservation (Fig. 4C), suggesting that cryopreservation might differentially affect the viability of specific subpopulations in EqMDEC cultures, including those responsible for the observed anti-cancer effects. Future experiments using single cell RNA sequencing to analyze non-CP and CP EqMDEC cultures may provide a more definitive answer to this conjecture.

Since our experiments with fractionated EqMDEC CM indicated that the bioactive factor(s) are small in size (Fig. 3A), we performed proteomics to characterize small peptides present in CM from CP and non-CP EqMDEC cultures. Mass spectrometry yielded a total of 288 peptides in non-CP EqMDEC CM and 448 peptides in the CP EqMDEC CM, of which 47 and 211 peptides, respectively, were specific to either non-CP or CP CM (Supplementary Table 1^1^). Interestingly, 12 out of the 47 peptides selectively present in the non-CP EqMDEC CM have a known role as tumor suppressors (Table 2), and PANTHER gene ontology analysis showed that these peptides were most commonly associated with 3 main pathways, whereas peptides selectively found in CP EqMDEC CM had a much more diverse profile (Supplementary Fig. 2A). However, none of the peptides selectively present in the non-CP EqMDEC CM had a clear role in cell death. This result drove us to focus on possible small molecules and, thus, untargeted metabolomics and lipidomics were performed to identify the potential bioactive factor(s) responsible for the anti-cancer effects observed in our system.

**Table 2:** Peptides with a known tumor suppressor function detected exclusively in fresh, non-cryopreserved eqMDEC conditioned medium (CM).

Accession	Description	# Peptides
1333610726	ADAMTS-like protein 1 isoform X1	6
545179509	C–C motif chemokine 7	3
169234968	C-X-C motif chemokine 10 precursor	3
221139848	C-X-C motif chemokine 2 precursor	2
825706118	C-X-C motif chemokine 6 precursor	8
1333704580	Extracellular sulfatase Sulf-1 isoform X1	4
349603351	Histidine triad nucleotide-binding protein 1-like protein	2
545217098	Histone H2B type 2-F	2
261490217	MHC class I antigen, partial	2
1333648020	Ribonuclease T2	2
1333694044	Thy-1 membrane glycoprotein	2
1333669017	Transforming growth factor beta receptor type 3 isoform X1	2

**Figure 5:**
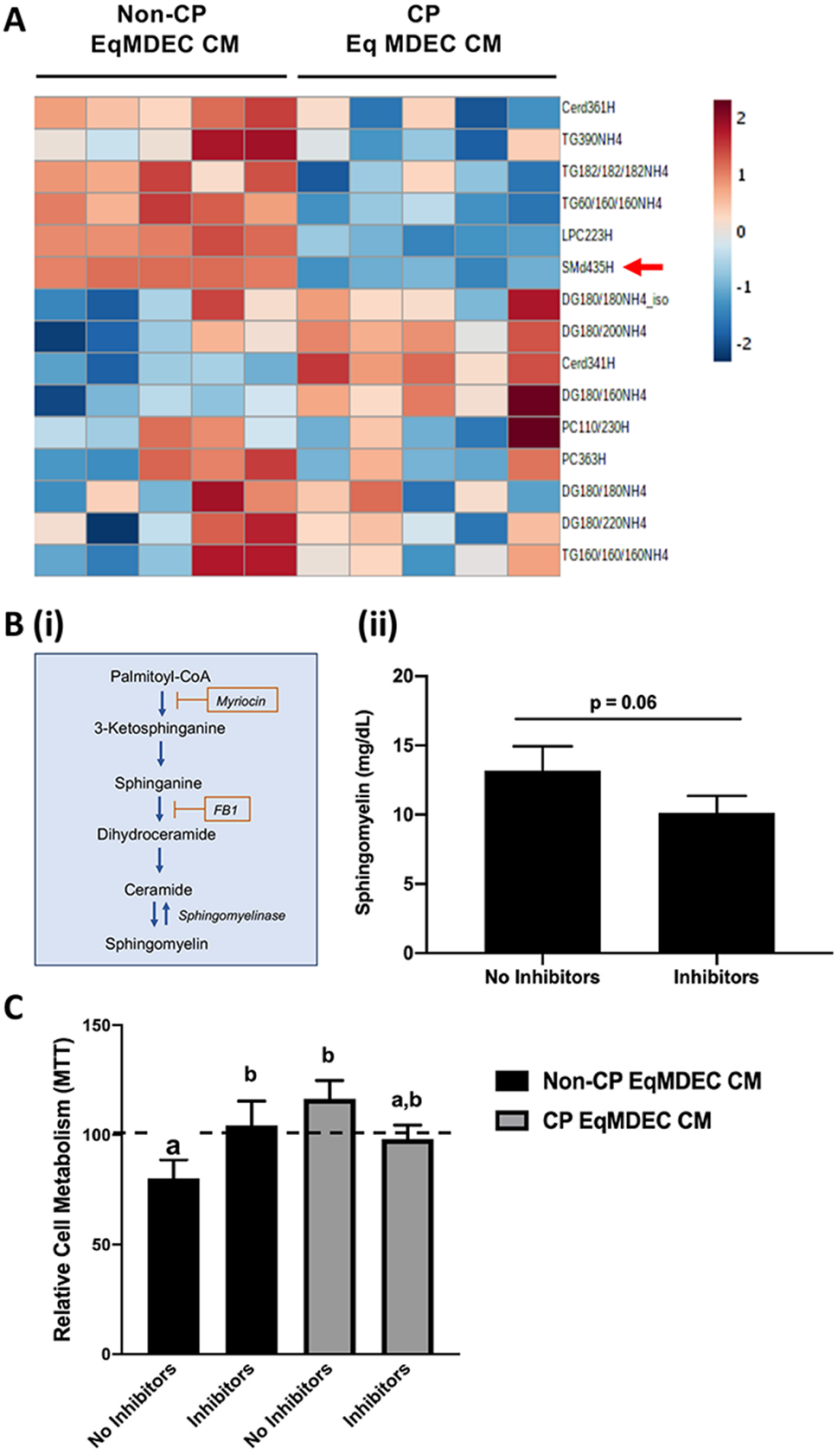
Sphingomyelins were found to mediate reduced cell viability of triple-negative human breast cancer (TNBC) cells. (**A**) Heat map showing lipid profiles of non-CP and CP EqMDEC CM. Red arrow indicates sphingomyelins. n = 5/group. (**B**) Simplified schematic overview of the sphingomyelin pathway, indicating where myriocin and fumosin B1 act to block sphingomyelin synthesis (i) and sphingomyelin concentration in CM collected from non-CP EqMDEC treated with or without sphingomyelin pathway inhibitors, as determined by a sphingomyelin assay kit (ii). (**C**) MTT assays of MDA-MB-231 cells cultured for 48 h with CM collected from non-CP and CP EqMDEC that were treated with or without sphingomyelin inhibitors. Asterisks depict significant differences: **p < 0.01. ns: not significant, n = 3. Data are presented as the mean ± standard deviation.

Metabolomics analysis detected over 100 metabolites in CM from EqMDEC isolated from 5 different animals, but without any clear pattern of metabolites that were both present in non-CP CM and absent in CP CM (Supplementary Fig. 2B). Global lipidomics analysis of these EqMDEC samples, however, yielded clear differences in the lipid profiles between non-CP and CP CM. Of particular interest were sphingomyelins (SM), which were detected at higher levels in the non-CP CM when compared to the CP CM (Fig. 5A). SM and their products, ceramides, are sphingolipids found in plasma membranes of cells as well as secreted in biological fluids. These secreted lipids can be taken up by cells in culture and have been implicated in apoptosis resulting in their potential as chemotherapeutic agents in disease, including breast cancer^16–18^. To determine if SM are involved in the anti-cancer effects of the EqMDEC secretome, we inhibited the SM pathway in non-CP EqMDEC using myriocin and fumosin B1 (Fig. 5B(i)). A clear reduction, albeit not reaching significance (p = 0.06), of SM in non-CP EqMDEC CM when treated with myriocin and fumosin B1 demonstrated the activity of these two inhibitors (Fig. 5B(ii)). Significantly less cell death was observed when MDA-MB-231 were cultured in CM from SM inhibitor-treated non-CP EqMDEC when compared to untreated non-CP EqMDEC CM, indicating that SM are involved in the anti-cancer properties of the EqMDEC secretome (Fig. 5C). As expected, no cell death was observed when MDA-MB-231 were cultured in CM from the same EqMDEC that were CP, and this did not change in the presence of SM inhibitors (Fig. 5C).

Collectively, these data identified SM in the equine MDEC secretome and confirmed the contribution of these lipids to cancer cell killing.

## Discussion

In this study, we show that mammosphere-derived cells from domesticated mammals with a low mammary cancer incidence, such as equines and bovines, secrete bioactivate factors that significantly decreased the viability of triple negative breast cancer (TNBC) cells in vitro and reduced tumorigenicity in a xenograft TNBC mouse model in vivo. Further in-depth analysis identified secreted sphingomyelins (SM) as contributors to this cancer cell killing. Based on these findings, we propose that secreted SM in the mammary gland could represent a key mediator involved in low mammary cancer incidence observed in certain mammals.

SM are plasma membrane sphingolipids present in most eukaryotic cells, notably in lipid rafts, where they play important roles in determining the biophysical properties of the cell membrane. The quantity and composition of SM varies greatly between different tissues and organs, and the physical properties of SM may vary based on acyl side chain composition. In addition, SM can be metabolized into the bioactive sphingolipids ceramide, sphingosine, and diacyglycerol, all of which play active roles in the sphingolipid cellular signaling network^19,20^.

SM have been an area of significant interest in cancer research^21^. For example, addition of SM to human pancreatic cancer cells was found to potentiate the chemotherapy drug gemcitabine via a mechanism of increased production of ceramide, mitochondrial depolarization, apoptosis, and cell death^22^. This study, as well as most others, evaluated the effects of SM on cancer progression by focusing on the ratio of SM to ceramides, which are known to regulate the apoptotic signaling pathway^23–25^. However, since our lipidomics analysis revealed no difference in ceramide species concentrations between CM from non-CP and CP EqMDEC, ceramide levels alone cannot account for the observed difference in cancer cell death upon exposure to non-CP versus CP EqMDEC CM. We propose another mechanism by which SM can display its anti-cancer effects in the mammary gland, namely via playing a biophysical role in lipid raft composition. SM are a major component of lipid rafts, which are specialized membrane microdomains that control the spatial organization of signaling molecules^26^, and changes in the composition of these lipid rafts are known to affect cell signaling^27,28^. For example, it has been reported that disrupting lipid rafts in TNBC cells, such as MDA-MB-231, via inhibition of another major component of lipid rafts, cholesterol, resulted in increased apoptosis and decreased expression of genes with anti-apoptotic roles^29^. This is consistent with our results, where MDA-MB-231 cells cultured with EqMDEC CM underwent cell death through apoptosis and showed downregulation of *CX3CL1*, *ASNS*, and *LCN2*, three genes associated with breast cancer progression. In addition, manipulation of lipid rafts in effector cells by secreted SM may also explain the selective killing of TNBC, but not ER^+^ or normal breast epithelial cells, as breast cancer cells with different receptor status have been found to have different lipid raft compositions^30^, and so, might respond differently to secreted SM. Interestingly, MDA-MB-231 cells produce more lipocalin 2 (LCN2) protein compared to MCF-7^31^, encoded by *LCN2* that was downregulated in MDA-MB-231 but not MCF-7, further adding to the rationale as to why the eqMDEC secretome preferentially affects this cancer cell type.

While most SM are confined to cell membranes, they can also be present in exosomes, which, together with microvesicles (MV), make up the general group of cell-secreted vesicles that are named extracellular vesicles (EV)^32^. This might explain the identification of SM in the EqMDEC CM in our present study. Interestingly, SM and other sphingolipids are not only involved in EV biogenesis, but contribute to EV action on target cells as well^33^. This raises the intriguing possibility that SM might not be directly responsible for the observed anti-cancer effects of the EqMDEC secretome, as discussed above, but that the enhanced presence of SM in EV secreted by EqMDEC increases the activity of EV cargo on the target cancer cells. It will, therefore, be of interest to determine in future experiments the composition and quantities of sphingolipid species in EV isolated from MDEC from mammals with a low versus high mammary cancer incidence. In a previous study, where we specifically focused on differences in protein cargo present in MV, our group identified differences in Wnt protein cargo in MV from MDEC from mammals with natural variation in mammary cancer incidence and how that resulted in altered signaling activity of the Wnt/β-catenin signaling pathways in these cells^34^. Based on our current findings, we see the importance of expanding this work to include exosomes and to evaluate how exosome-associated sphingolipids affect the delivery and activity of EV cargo on target cells.

In addition to their basic roles in cell structure, signaling, and EV biogenesis, lipids have also been shown to play important roles in the overall health of organisms, such as influencing lifespan. Indeed, there is increasing evidence that variation in lipid profiles may contribute to endogenous differences in longevity, which may be achieved by both lineage-specific adaptations and common mechanisms across species^35,36^. For example, one study analyzed the concentrations of more than 20,000 lipid compounds in five different tissues (liver, muscle, kidney, heart, and brain) across three mammalian clades (primates, rodents, bats). Several classes of lipids, including triacylglycerol, glycerophospholipids, and sphingolipids, were shown to have coordinated concentration changes in different tissues among the long-living species of each clade^37^. Interestingly, many of these long-lived mammals also display cancer resistance mechanisms. A better understanding of the molecular mechanisms underlying and/or linking lifespan regulation and cancer resistance could be used to develop approaches to modulate these two processes, which are of central importance to human health care^1,2^.

In summary, our study contributes to the growing list of protective mechanisms identified in species with no or low cancer incidence. A better understanding of the complex regulatory mechanisms of SM specifically, and lipids in general, in the mammary gland will improve our understanding of cell death regulation and cancer resistance/susceptibility in this unique organ, and may eventually lead to new avenues for prevention and treatment of breast cancer.

## Methods

### Animal welfare

All experimental protocols for the collection of mammary gland tissues were approved by the ethics committee/Institutional Animal Care and Use Committee of Cornell University, and all methods were carried out in accordance with relevant guidelines and regulations. Samples were collected after euthanasia only, and all animals were research, not client-owned, animals. For the in vivo mice experiments, all methods were carried out in accordance with relevant guidelines and regulations, and were approved by the Institutional Animal Care and Use Committees (IACUC) at Cornell University (#2013-0022).

### Isolation and growth of mammosphere-derived epithelial cells (MDEC)

Mammary gland tissues from clinically healthy, non-lactating, research mares (4–20 years old) and slaughterhouse heifers (3–5 years old), euthanized for reasons unrelated to this study, were collected by excising 2 pieces of 5 cm^2^ of tissue next to the median line of the mammary gland compartments. Canine mammary gland samples were collected from healthy, non-lactating, research beagles (6–10 years old), euthanized for reasons unrelated to this study, by excising at least 2 cm^2^ of tissue near the nipple. Samples were processed to establish and maintain MDEC cultures, exactly as described before^11,38,39^. Mammary fibroblasts, isolated from the same mammary tissues to provide tissue-matched cells, were cultured in cell line medium, consisting of Dulbecco’s modified Eagle medium (DMEM) supplemented with 10% fetal bovine serum (FBS) and 1% penicillin/ streptomycin (P/S) (cell line medium).

### Generation of conditioned medium (CM) and manipulation

CM was collected from MDEC or mammary fibroblasts after 2 days of culture, when cells were 70% confluent. To this end, 1 × 10^6^ cells were seeded in a T75 flask with 10 mL of DMEM + 10% FBS. After 24 h, medium was changed to DMEM without serum, unless when indicated otherwise. Medium was collected 48 h later, centrifuged twice for 10 min at 300×*g* to remove any cellular debris, and used for further experiments. CM from human mammary cell lines was used as a control (self-CM) and was collected as described above. To generate CM from MDEC in which the sphingomyelin pathway was blocked, 0.5 µg/ml myriocin and 0.25 µg/ml fumosin (Sigma, St. Louis, MO) was added to the DMEM without serum. CM was collected 48 h later, as described above. Sphingomyelin concentrations in CM were assessed using a sphingomyelin assay kit (Cell Biolabs, San Diego, CA), as per manufacturer’s instructions.

To determine the nature of bioactive factors in the EqMDEC secretome, CM was fractionated using centrifugal filter devices with different Nominal Molecular Weight Limit (NMWL) Ultracel membranes (Sigma). To this end, 5 ml of EqMDEC CM was loaded onto filter devices with Ultracel membranes ranging from 3 to 100 kDA, and centrifuged according to manufacturer’s instructions. The solute was then re-suspended in the same volume of DMEM as the loaded sample and used for viability assays, as described below. Alternatively, CM was heated to 100 °C for 10 min or frozen at − 80 °C for one week, then brought to RT, and used for viability assays. Lyophilized CM was reconstituted with sterile water to the original volume before use in assays. Finally, CM was treated with 1U/ml proteinase K and incubated at 37 °C for 6 h. Treated CM was placed on ice for 10 min, then brought to RT, and used for viability assays.

### Human mammary cell lines

All human cell lines were obtained from ATCC. The human normal breast epithelial cell line MCF10A was cultured in DMEM/F12 supplemented with 5% horse serum, 1% P/S, 10 μg/ml human insulin 20 ng/ml epidermal growth factor (EGF) and 0.5 μg/ml hydrocortisone (all from Sigma). The estrogen receptor (ER)-positive breast cancer cell lines MCF7 and BT474 and the triple negative breast cancer (TNBC) cell lines MDA-MB-231 and MDA-MB-468 were cultured in cell line medium. The TNBC cell line Hs578T was cultured in DMEM supplemented with 0.01 mg/mL bovine insulin, 10% FBS, and 1% P/S.

### Cell viability assays

After 48 h of culturing human mammary cell lines in CM, cell line medium, or DMEM, a 3-(4,5-dimethylthiazol-2-yl)-2,5-diphenyltetrazolium bromide (MTT) in vitro toxicology assay or a lactate dehydrogenase (LDH) assay was carried out, as previously described^11^, according to manufacturer’s instructions (Sigma). MTT and LDH absorbances were measured at 570 nm and 490 nm, respectively, on a Multiskan EX plate reader (Thermo Scientific, Vantaa, Finland) and background measurements of 690 nm were subtracted for both. Optical densities of wells treated with EqMDEC CM were compared to those treated with either DMEM or control (self-CM) in order to determine cell viability or relative LDH release. Values were expressed relative to wells treated with DMEM and to lysed wells for MTT and LDH release, respectively. For active caspase 3 immunostaining, 4% paraformaldehyde-fixed cells were washed with PBS and treated with 0.5% Triton X-100 for 10 min. Following a 30 min incubation in blocking solution (1% goat serum and 1% BSA in PBS) at RT, fixed cultures were probed with an anti-active caspase 3 primary antibody diluted 1:100 in PBS (ab4051, Abcam, Cambridge, MA) for 1 h, followed by incubation with HRP-conjugated goat anti-rabbit diluted 1:100 in PBS (Jackson ImmunoResearch, West Grove, PA) for 1 h.

### Animal studies

Age-matched female NOD scid gamma (NSG) mice between 6–8 weeks old (The Jackson Laboratory, Bar Harbor, ME) were used for xenograft studies. Ten NSG mice (5 controls and 5 treated) were injected subcutaneously with 3 × 10^6^ MDA-MB-231 cells into the left 4^th^ mammary gland. Cohort size and cell numbers were chosen based on published work describing xenograft experiments with MDA-MB-231 cells that relied on similar read-outs^40,41^. Fifteen days after cell injection, mice were randomly sorted into two groups and injected intratumorally with 150 μl EqMDEC CM or DMEM (control) daily for two weeks, after which mice were sacrificed and tumors were removed and measured. Tumor diameter was measured daily by digital caliper, and tumor volume (mm^3^) was calculated using the formula: a^2^ x b/2, where “a” is the shortest diameter and “b” is the longest diameter of the tumor. The measurements were taken by researchers who were blinded to avoid bias.

### Flow cytometric analyses

Procedures were followed, as previously described^35^. Briefly, EqMDEC were collected using Accutase and stained with anti-CD44 diluted 1:20 (553133, BD Biosciences, San Jose, CA), anti-CD49f diluted 1:10 (FAB135OU, Novus, Centennial, CO), or anti-CD29 diluted 1:10 (CBL481,Sigma) antibodies, in PBS with 1% BSA for 1 h. Staining with an isotype control antibody, or no antibody stain were included as controls. Cells were washed three times in PBS and incubated with an Alexa-488 conjugated secondary goat anti-mouse antibody (Jackson ImmunoResearch) diluted in PBS with 1% BSA, for 30 min where appropriate. After washing, 50,000 cells were analyzed on a Gallios flow cytometer (Beckman Coulter, USA). Data analysis was conducted using Kaluza Flow Cytometry Analysis software version 1.5 (https://www.beckman.com/flow-cytometry/software/kaluza).

### RNA deep sequencing

Procedures were followed, as previously described^11^. Briefly, MDA-MB-231 and MCF7 were cultured for 24 h prior to a 12 h treatment with self-CM or EqMDEC CM. Total RNA was extracted with TRIzol reagent following manufacturer’s recommendations (Sigma). The quality of total RNA was evaluated using the Agilent 2100 bioanalyzer with the RNA 6000 Nano LabChip kit (Agilent, Santa Clara, CA). RNA-seq libraries were prepared with the NEBNext Ultra Directional RNA Library Prep Kit (NEB, Ipswich, MA) using 500 ng total RNA followed by polyA + enrichment, and were sequenced using Illumina NextSeq500 to obtain 81 nucleotide (nt) single-end reads. The reads were trimmed to remove adaptor and low-quality bases with cutadapt v1.8.3, aligned with TopHat 2.1.1 (https://ccb.jhu.edu/software/tophat/index.shtml), and then analyzed for differential gene expression using cuffdiff v2.2.1 2.2.1 (http://cole-trapnell-lab.github.io/cufflinks/releases/v2.1.1/) using Ensembl annotations. Transcript abundance was measured in fragments per kilo base (kb) of exon per million fragments mapped (FPKM).

### Gene expression analyses

Procedures were followed, as previously described^11^. Briefly, MDA-MB-231 were seeded at a density of 2 × 10^5^ in T25 tissue culture flasks. After 24 h, culture medium was removed, cell monolayers were rinsed with PBS, and cells were incubated in either self-CM or EqMDEC CM for 24 h. Subsequently, mRNA was extracted from the cells using a RNeasy Plus Kit (QIAGEN, Germantown, MD) and cDNA was synthesized using M-MLV Reverse Transcriptase (Promega, Madison, WI) both according to manufacturer’s protocols. SYBER green-based (Bio-Rad, Hercules, CA) reverse transcriptase polymerase chain reaction (qRT-PCR) assays were carried out on an Applied Biosystems 7500 Fast Real Time PCR instrument to determine fold changes in gene expression. The comparative Ct method was used to quantify gene expression levels where ΔΔCt = ΔCt (sample) – ΔCt (reference). The reference gene Glyceraldehyde 3-phosphate dehydrogenase (*GAPDH*) was used to normalize samples. Primers to amplify *PPFIBP2, RARRES2, CX3CL1, ASNS, LCN2* and the housekeeping gene *GAPDH,* were designed using Primer3 software version 0.4.0 (http://bioinfo.ut.ee/primer3-0.4.0/), based on the human sequence found in the National Center of Biotechnology Information (NCBI) GenBank. All samples were run in triplicate.

### Multi-omics analysis

Proteomics, metabolomics, and lipidomics, were performed on CM collected from cell culture-matched EqMDEC that were either freshly isolated (non-cryopreserved, non-CP) or re-cultured after cryopreservation (CP), based on previously published work with slight modifications^42–44^. A detailed description of the procedures is provided in the Supplementary Methods.

### Statistical analyses

Data were obtained from at least three independent experiments, each using MDEC from three different individuals, and expressed as the mean ± standard deviation for each group. Five mice per group were used for in vivo experiments and the injected eqMDEC CM was from one individual horse collected at different passages. Statistical analyses, including Student’s t-test and one-way analysis of variance, were performed using GraphPad Prism software version 8.3.1 (https://www.graphpad.com) (GraphPad, Inc., La Jolla, CA, USA). P < 0.05 was considered to indicate a statistically significant difference.

## Supplementary information

Supplementary Information.

## Data Availability

The raw sequencing data are available for download from the Gene Expression Omnibus (http://www.ncbi.nlm.nih.gov/geo/) under accession number #GSE145536.
