# Supplementary material for: Secreted sphingomyelins modulate low mammary cancer incidence observed in certain mammals
Source: Sci Rep. 2020 Nov 25;10:20580. doi: 10.1038/s41598-020-77639-1 (PMC7689471; doi:10.1038/s41598-020-77639-1)
Supplement: Supplementary file 1 — Supplementary Information. [file 41598_2020_77639_MOESM1_ESM.pdf]

Supplementary Information for:

Secreted sphingomyelins modulate low mammary cancer incidence observed in certain mammals

Melissa M. Ledet, Rebecca M. Harman, Jennifer C. Fan, Emily Schmitt-Matzen, Maria Elena Diaz-Rubio, Sheng Zhang, Gerlinde R. Van de Walle

Corresponding author: Gerlinde R. Van de Walle; Email: grv23@cornell.edu

This pdf file includes:

- Supplementary Methods
- Supplementary Figures S1 and S2
- Supplementary Table 1

SUPPLEMENTARY INFORMATION on -OMICS METHODS

Proteomics. In-solution digestion was performed following a protocol by Zhang et al. (2003) with slight modifications (1). The protein pellet from a 10 mL medium culture was dissolved in 300 µl denaturing solution containing 6.0 M guanidine-hydrochloride, and 100 mM PBS pH 7.0, sonicated for 5 min, centrifuged, and supernatant was collected. The denatured sample was reduced with 10 mM dithiothreitol and alkylated with 55 mM iodoacetamide for 1 h at RT. Excess iodoacetamide was quenched with 30 mM dithiothreitol for 30 min at RT. The samples were diluted to a final volume of 3,775 µl with 50mM Tris hydrochloride pH 8.0 to reduce the guanidine-hydrochloride concentration to less than 1M. The samples were digested by adding 25 µg trypsin (Promega, Madison, WI) and incubated at 37 ºC for 16 h. Digestions were stopped by the addition of 25 µl trifluoroacetic acid. The digests were further desalted by solid phase extraction using Sep-Pack Cartridge (Waters Corporation, Milford, MA), and eluted peptides were evaporated to dryness by a Speedvac SC110 (Thermo Savant, Milford, MA) prior to nanoLC-MS/MS analysis on an Orbitrap Fusion platform, as previously described (5). The subsequent data-dependent acquisition raw files were subjected to database searches using Proteome Discoverer 2.2 software (https://www.thermofisher.com/us/en/home/industrial/mass-spectrometry/liquid-chromatography-mass-spectrometry-lc-ms/lc-ms-software/multi-omics-data-analysis/proteome-discoverer-software.html) (ThermoFisher Scientific, Bremen, Germany) with the Sequest HT algorithm, as reported previously (2).

Metabolomics. For untargeted metabolomics analysis, each of the cell media samples (200 µl) along with a mixed sample for QC (quality control) containing an equal amount of each sample was evaporated to dryness under vacuum. The dried samples were reconstituted in 100 µl of 50% acetonitrile (ACN) with 0.1% formic acid for subsequent revere phase liquid chromatography

(RPLC) analysis, and in 100 µl of 60% ACN for hydrophilic interaction liquid chromatography (HILIC) analysis. Subsequent LC-MS and LC-MS/MS data acquisition was conducted on a Vanquish UHPLC coupled to a Q Exactive Hybrid Quadrupole-Orbitrap High Resolution Mass Spectrometer (Thermo Fisher Scientific, San Jose, CA, USA). For RPLC-MS analysis, an Accucore Vanquish C18+, 1.5µm column (2.1 mm id x 100 mm) was used. The mobile phase consisted of (A) 0.1% formic acid and (B) acetonitrile with 0.1% formic acid. The gradient was as follows: 0 –2.0 min (0.5 - 1% solvent B), 2.0–6.5 min (1–20% B), 6.5-11.5 min (20-95% B), 11.5-13.5 min (95-99% B), 13.5-16.5min (99-100% B), 16.5-19 min (100-0.5% B), 19-24 min (0.5% B). The flow rate was 320 µl/min and 2 µl of each sample were injected. For HILIC-MS analysis, a SeQuant ZIC pHILIC column (5um, 2.1 x 150 mm) was used. The mobile phase consisted of (A) 10 mM ammonium acetate pH 9.8 and (B) acetonitrile. The gradient was as follows: 0-15 min, 90-30% solvent B; 15-18 min, 30% B; 18-19 min, 30–90% B; 19-27 min, 90% B; followed by a 3-min of re-equilibration of the column before next run. The flow rate was 250 µl/min and 2 µl of each sample were injected. To avoid possible bias, the sequence of sample injections was randomized for different samples and their biological replicates. All of the samples in both RPLC-MS and HILIC-MS analyses were acquired using both positive and negative electrospray ionization analyses in full scan MS mode for relative quantitation as well as data dependent MS/MS mode on the QC samples for compound identifications. Nitrogen as sheath, auxiliary, and sweep gas was set at 50, 8, and 1, arbitrary units, respectively. The QE-HF system was operated with spray voltage at 3.50 kV; and capillary temperature at 275 °C. MS survey scans were acquired at a resolving power of 120,000 (fwhm at m/z 200) for the mass range of m/z 67-1000 with automatic gain control target at 3e6 ions and maximum injection time at 100 ms. Data-dependent MS-MS spectra for top 15 most intensity ions after each full MS scan were generated for QC pool

samples using the following parameters: resolving power at 15,000; automatic gain control target at 1e5 ions; maximum injection time for 50 ms; isolation window at 0.4 m/z; and the step normalized collision energy of 20, 30, 40%. All data were acquired under Xcalibur 4.0 operation software (https://www.thermofisher.com/order/catalog/product/OPTON-30965#/OPTON-30965) (Thermo-Fisher Scientific). The acquired datasets composed of full MS and data-dependent MS-MS raw files, were processed using Compound Discoverer 3.1 (https://www.thermofisher.com/us/en/home/industrial/mass-spectrometry/liquid-chromatography-mass-spectrometry-lc-ms/lc-ms-software/multi-omics-data-analysis/compound-discoverer-software.html). An untargeted metabolomics workflow with putative identification through ChemSpider (http://www.chemspider.com) and mzCloud (https://www.mzcloud.org) databases were used for processing the raw data. The software parameters for alignment were 5 ppm mass tolerance for the adaptive curve model and 0.5 min maximum shift for alignment. The software parameters for detecting unknown compounds were 5 ppm mass tolerance for detection, 30% intensity tolerance, 3 for the sensitivity and noise threshold, and 2×10^6 minimum peak height.

Lipidomics. Stock lipid standards were prepared by dissolving them in dichloromethane (DCM)/methanol (MeOH; 2:1 v/v) at concentration ranging 1 to 5 mg/ml, which were stored at -20°C. An internal standard mixture used for spiking into each sample prior to extraction consisted of 25 μg/mL of following lipids: trygliceride (TG) 15:0/15:0/15:0 (Sigma-Aldrich, St. Louis, MO), phosphatidyl choline (PC) 17:0/17:0 (Avanti Polar Lipids, Alabaster, AL), phosphatidylglycerol (PG) 14:0/14:0 (Avanti Polar Lipids), lysophosphatidyl-choline (LPC) 20:0 (Avanti Polar Lipids), ceramide d18:1/17:0 (Avanti Polar Lipids) and cholesteryl ester 17:0 (Avanti Polar Lipids). Sample preparation was conducted as previous reported (3), with slight modifications.

The internal standard mixture (30 µl) was spiked to 30 µl sample, to which 190 µl of MeOH was added. Samples were then vortexed for 20 sec prior to addition of 380 µl of dichloromethane (DCM). The resulting samples were vortexed for 20 sec, and then 120 µl of water was added to induce phase separation. The samples were then vortexed for 10 sec and allowed to equilibrate at RT for 10 min before centrifugation at 8,000 × g for 10 min at 10 °C. A total of 370 µl of the lower lipid-rich DCM layer was then collected and the solvent was evaporated to dryness under vacuum. Samples were reconstituted in 150 µl of acetonitrile (ACN)/2-propanol (IPA)/water (65:30:5 v/v/v) for subsequent chromatographic separation. Chromatographic separation was performed on a Vanquish UHPLC system with an Accucore C30, 2.6 µm column (2.1 mm id x 150 mm) coupled to a QE-HF Mass Spectrometer (Thermo Fisher Scientific, San Jose, CA). The mobile phase consisted of (solution A) 60% ACN, 40% water, 10 mM ammonium formate with 0.1% formic acid and (solution B) 90% IPA, 10% ACN, 10 mM ammonium formate with 0.1% formic acid. The gradient was as follows: 0-1.5 min, 32% solvent B; 1.5-4 min,32-45% B; 4-5 min, 45-52% B; 5-8 min 52-58% B; 8-11 min, 58-66% B; 11-14 min, 66-70% B; 14-18 min, 70-75% B; 18-21 min, 75-97%s B; 21-25 min, 97% B; 25-25.1 min 97-32% B; followed by a 4-min of re-equilibration of the column before next run. The flow rate was 260 µl/min and 2 µl of each sample were injected. To avoid possible bias, the sequence of sample injections was randomized for different samples and their biological replicates. All of the samples were analyzed by positive electrospray ionization in data-dependent MS-MS mode. The QE-HF setting was the same as described above for untargeted metabolomics, except of 5 arbitrary units used for auxiliary gas and the step normalized collision energy of 25 and 35% used for lipidomics. Acquired MS/MS data was processed using LipidSearch™ software version 4.1 (https://www.thermofisher.com/us/en/home/industrial/mass-spectrometry/liquid-chromatography-mass-spectrometry-lc-ms/lc-ms-software/multi-omics-data-

analysis/lipid-search-software.html) (Thermo Scientific) with the following workflow: First, the individual data files were searched for product ion MS/MS spectra of lipid precursor ions. MS/MS fragment ions were predicted for all precursor adduct ions measured within ± 5 ppm. The product ions that matched the predicted fragment ions within a ± 5 ppm mass tolerance were used to calculate a match-score, and those candidates providing the highest quality match were determined. Next, the search results from the individual positive ion files from each sample group were aligned within a retention time window (± 0.1 min) and the data were merged for each annotated lipid. Lipids were then normalized by class using the internal standard mix added before the extraction. The annotated lipids were then filtered to further reduce false positives using the criteria as listed on the following inserted Table:

Filter Criteria	Lipid class
Main ion= M+H or M-HCOO	PC, LPC
Main ion= M+H or M+HCOO	Cer, CerG
Main ion= M+H	LSM, SM, So
Main ion= M+NH4	MG, DG, TG, Che
Main ion= M+H or M-H	PE, LPE, PS, LPS
Main ion= M+NH4 or M-H	PI, PG, LPI, LPG
Main ion= M-H	PA, LPA, DLCL, MLCL
Main ion= M-2H or M-H	CL
Main grade[a] = A, B, C,	ALL

Main grade[a]: A: Both lipid class and fatty acid were completely identified; B: Lipid class and some of the fatty acid were identified; C: Either lipid class or the fatty acid was identified.

References

1. S. Zhang, C. K. Van Pelt, J. D. Henion. Automated chip-based nanoelectrospray-mass spectrometry for rapid identification of proteins separated by two-dimensional gel electrophoresis. *Electrophoresis* **24**, 3620-3632 (2003).

2. R. M. Harman, M. K. He, S. Zhang, G. R. Van de Walle. Plasminogen activator inhibitor-1 and tenascin-C secreted by equine mesenchymal stromal cells stimulate dermal fibroblast migration in vitro and contribute to wound healing in vivo. *Cytotherapy* **20**, 1061-1076 (2018).

3. S. S. Bird, V. R. Marur, M. J. Sniatynski, H. K. Greenberg, B. S. Kristal. Serum lipidomics profiling using LC-MS and high-energy collisional dissociation fragmentation: focus on triglyceride detection and characterization. *Anal. Chem.* **83**, 6648-6657(2011).

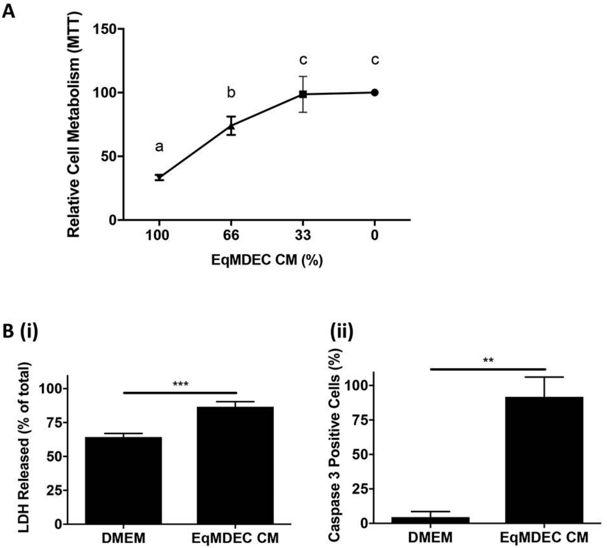

Supplementary Figure 1. Mammosphere-derived epithelial cell conditioned medium (MDEC CM) causes dose-dependent cell death of MDA-MB-231 cells by apoptosis. (A). MTT assays of MDA-MB-231 cells cultured for 48 h with varying concentrations of equine (Eq) MDEC CM. **(B).** Lactate dehydrogenase (LDH) assay **(i)** and quantification of activated caspase-3-positive cells **(ii)** of MDA-MB-231 cells after 48 h culture with DMEM (control) or EqMDEC CM. Significant differences are either depicted by asterisks: $^{**}p<0.01$, $^{***}p<0.001$, or by different letters. n=3. Data are presented as the mean ± standard deviation.

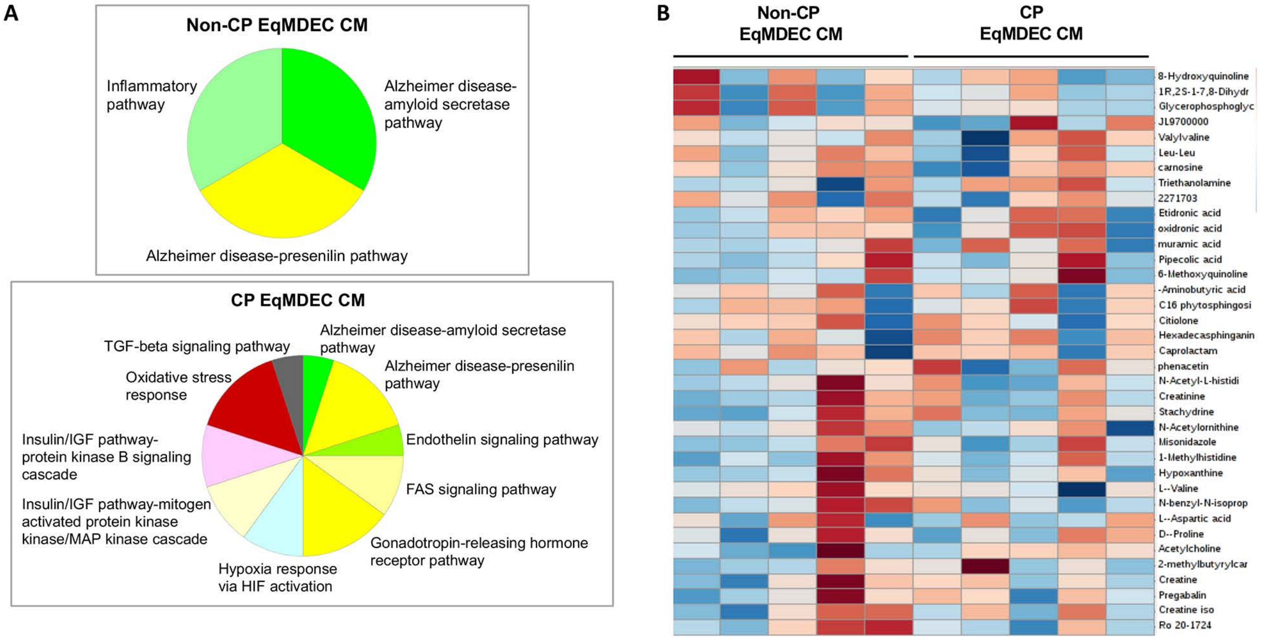

Supplementary Figure 2. Non cryopreserved (non-CP) equine mammosphere-derived epithelial cell conditioned medium (EqMDEC CM) has a peptide profile highly enriched in inflammatory pathways and a metabolite profile that is not clearly different compared to that of cryopreserved (CP) eqMDEC CM. (A). PANTHER-generated pie charts of the pathways represented by peptides secreted by non-CP and CP EqMDEC as identified by mass spectrometry. n=1/group. **(B).** Heat map showing metabolite profiles of non-CP and CP EqMDEC CM. n=5/group.

Supplementary Table 1. Peptides present in fresh (non-CP) and cryopreserved (CP) eqMDEC conditioned medium (CM) using mass spectrometry.

non-CP eqMDEC CM			CP eqMDEC CM		
**Accession**	**Description**	**# Peptides**	**Accession**	**Description**	**# Peptides**
149724150	14-3-3 protein epsilon isoform X1 [Equus caballus]	3	1333587315	10 kDa heat shock protein, mitochondrial isoform X2 [Equus caballus]	4
1333569388	14-3-3 protein gamma [Equus caballus]	2	1333617600	14 kDa phosphohistidine phosphatase isoform X9 [Equus caballus]	2
1333596281	45 kDa calcium-binding protein [Equus caballus]	6	1333607386	14-3-3 protein beta/alpha [Equus caballus]	4
1333587311	60 kDa heat shock protein, mitochondrial [Equus caballus]	2	149724150	14-3-3 protein epsilon isoform X1 [Equus caballus]	5
1333565115	60S acidic ribosomal protein P2 isoform X1 [Equus caballus]	2	1333569388	14-3-3 protein gamma [Equus caballus]	6
1333632148	72 kDa type IV collagenase [Equus caballus]	31	149727776	14-3-3 protein theta [Equus caballus]	2
1333616111	78 kDa glucose-regulated protein [Equus caballus]	13	149721580	14-3-3 protein zeta/delta [Equus caballus]	4
1333572612	A disintegrin and metalloproteinase with thrombospondin motifs 2 isoform X1 [Equus caballus]	2	149722119	26S proteasome regulatory subunit 6B [Equus caballus]	2
1333549750	A disintegrin and metalloproteinase with thrombospondin motifs 7 isoform X1 [Equus caballus]	5	1333600710	40S ribosomal protein S10 [Equus caballus]	2
1333552051	actin, alpha cardiac muscle 1 [Equus caballus]	10	149757872	40S ribosomal protein S11 [Equus caballus]	2

1333568388	actin, cytoplasmic 1 isoform X1 [Equus caballus]	13	827834182	40S ribosomal protein S12 [Equus caballus]	2
1333610726	ADAMTS-like protein 1 isoform X1 [Equus caballus]	6	953859656	40S ribosomal protein S13 [Equus caballus]	2
1333652367	adipocyte enhancer-binding protein 1 isoform X1 [Equus caballus]	10	1333698817	40S ribosomal protein S15 [Equus caballus]	2
255522895	ADM precursor [Equus caballus]	3	149722116	40S ribosomal protein S16 [Equus caballus]	2
1333596351	agrin isoform X6 [Equus caballus]	31	149722214	40S ribosomal protein S17 [Equus caballus]	3
1333612548	alpha-actinin-1 isoform X4 [Equus caballus]	4	1333608695	40S ribosomal protein S21 [Equus caballus]	3
1333554253	alpha-actinin-4 isoform X6 [Equus caballus]	8	545221014	40S ribosomal protein S28 [Equus caballus]	2
338720674	amyloid-beta A4 protein isoform X1 [Equus caballus]	13	953847512	40S ribosomal protein S3a [Equus caballus]	3
1333544280	annexin A2 isoform X1 [Equus caballus]	16	1333542240	40S ribosomal protein S4, X isoform [Equus caballus]	4
1333597804	annexin A5 isoform X1 [Equus caballus]	4	149736868	40S ribosomal protein S6 [Equus caballus]	2
953851910	antithrombin-III [Equus caballus]	4	1333579684	40S ribosomal protein S7 [Equus caballus]	7
306922408	ATP synthase subunit beta, mitochondrial [Equus caballus]	2	194207535	40S ribosomal protein S8 [Equus caballus]	3
1333594974	basement membrane-specific heparan sulfate proteoglycan core protein isoform X6 [Equus caballus]	98	1333596281	45 kDa calcium-binding protein [Equus caballus]	5
1333610968	beta-1,4-galactosyltransferase 1 [Equus caballus]	5	1333587311	60 kDa heat shock protein, mitochondrial [Equus caballus]	12

28315888	beta-2-microglobulin [Equus caballus]	7	1333565115	60S acidic ribosomal protein P2 isoform X1 [Equus caballus]	5
1333596460	bone morphogenetic protein 1 isoform X1 [Equus caballus]	14	1333600861	60S ribosomal protein L10a [Equus caballus]	4
1333585664	BTB/POZ domain-containing protein KCTD12 [Equus caballus]	2	194225847	60S ribosomal protein L12 [Equus caballus]	4
545179509	C-C motif chemokine 7 [Equus caballus]	3	1333582755	60S ribosomal protein L14 [Equus caballus]	2
1333560984	C-type mannose receptor 2 isoform X1 [Equus caballus]	6	306922364	60S ribosomal protein L18 [Equus caballus]	2
169234968	C-X-C motif chemokine 10 precursor [Equus caballus]	3	149730048	60S ribosomal protein L21 [Equus caballus]	2
221139848	C-X-C motif chemokine 2 precursor [Equus caballus]	2	194208116	60S ribosomal protein L22 [Equus caballus]	2
825706118	C-X-C motif chemokine 6 precursor [Equus caballus]	8	149731349	60S ribosomal protein L24 [Equus caballus]	5
1333702207	cadherin-2 [Equus caballus]	9	1333573060	60S ribosomal protein L26-like 1 isoform X2 [Equus caballus]	3
146149237	calcitonin gene-related peptide 1 isoform CGRP preproprotein [Equus caballus]	2	149720018	60S ribosomal protein L27a [Equus caballus]	2
126352532	calcitonin gene-related peptide 2 precursor [Equus caballus]	2	149721564	60S ribosomal protein L30 [Equus caballus]	5
1333613646	calmodulin-1 [Equus caballus]	4	338720515	60S ribosomal protein L35 [Equus caballus]	2
1333595727	calsyntenin-1 isoform X1 [Equus caballus]	6	545217268	60S ribosomal protein L5 isoform X1 [Equus caballus]	3
149705852	calumenin isoform X1 [Equus caballus]	3	1333617194	60S ribosomal protein L7a [Equus caballus]	2

545209689	cathepsin B isoform X1 [Equus caballus]	10	1333632148	72 kDa type IV collagenase [Equus caballus]	32
1333568074	cathepsin D [Equus caballus]	2	1333616111	78 kDa glucose-regulated protein [Equus caballus]	25
1333609553	cathepsin L1 isoform X2 [Equus caballus]	15	1333619362	A disintegrin and metalloproteinase with thrombospondin motifs 1 [Equus caballus]	2
149731345	CD166 antigen isoform X1 [Equus caballus]	4	1333549750	A disintegrin and metalloproteinase with thrombospondin motifs 7 isoform X1 [Equus caballus]	2
338714429	cell growth regulator with EF hand domain protein 1 [Equus caballus]	4	1333649455	A-kinase anchor protein 12 isoform X1 [Equus caballus]	2
1279526421	Chain A, Serum albumin	39	1333588617	actin-related protein 3 [Equus caballus]	4
827834423	chemokine (C-X-C motif) ligand 1 (melanoma growth stimulating activity, alpha) precursor [Equus caballus]	4	1333552051	actin, alpha cardiac muscle 1 [Equus caballus]	20
1333567776	chitinase domain-containing protein 1 isoform X2 [Equus caballus]	2	149689874	actin, aortic smooth muscle [Equus caballus]	19
545195240	chondroitin sulfate proteoglycan 4 [Equus caballus]	2	1333568388	actin, cytoplasmic 1 isoform X1 [Equus caballus]	19
953892806	chordin-like protein 1 isoform X3 [Equus caballus]	9	149694095	adenylate kinase 2, mitochondrial isoform X1 [Equus caballus]	2
1048721	clusterin [Equus caballus]	19	545208028	adenylyl cyclase-associated protein 1 isoform X1 [Equus caballus]	2
149725588	cofilin-1 [Equus caballus]	4	1333652367	adipocyte enhancer-binding protein 1 isoform X1 [Equus caballus]	5
1333552835	cofilin-2 isoform X1 [Equus caballus]	2	255522895	ADM precursor [Equus caballus]	2

545192605	coiled-coil domain-containing protein 80 [Equus caballus]	6	1333596341	agrin isoform X1 [Equus caballus]	6
1333562231	collagen alpha-1(I) chain [Equus caballus]	103	1333657683	aldose reductase [Equus caballus]	2
643431288	collagen alpha-1(I), partial [Equus caballus]	5	1333639721	alpha-2-macroglobulin receptor-associated protein [Equus caballus]	2
1333586033	collagen alpha-1(IV) chain [Equus caballus]	5	1333612548	alpha-actinin-1 isoform X4 [Equus caballus]	20
1333617313	collagen alpha-1(V) chain isoform X1 [Equus caballus]	23	1333554253	alpha-actinin-4 isoform X6 [Equus caballus]	29
1333617315	collagen alpha-1(V) chain isoform X2 [Equus caballus]	20	338720674	amyloid-beta A4 protein isoform X1 [Equus caballus]	13
194226345	collagen alpha-1(VI) chain [Equus caballus]	43	1333544280	annexin A2 isoform X1 [Equus caballus]	23
1333556707	collagen alpha-1(XII) chain isoform X1 [Equus caballus]	32	1333597804	annexin A5 isoform X1 [Equus caballus]	9
1333618603	collagen alpha-1(XV) chain isoform X2 [Equus caballus]	5	1333573487	annexin A6 isoform X1 [Equus caballus]	3
1333620681	collagen alpha-1(XVIII) chain isoform X1 [Equus caballus]	13	953851910	antithrombin-III [Equus caballus]	4
149705490	collagen alpha-2(I) chain [Equus caballus]	93	545224964	ATP synthase subunit alpha, mitochondrial [Equus caballus]	6
1333586031	collagen alpha-2(IV) chain [Equus caballus]	7	306922408	ATP synthase subunit beta, mitochondrial [Equus caballus]	10
149730792	collagen alpha-2(V) chain [Equus caballus]	48	545178265	ATP synthase subunit d, mitochondrial isoform X1 [Equus caballus]	2
1333620786	collagen alpha-2(VI) chain isoform X1 [Equus caballus]	29	1333566994	barrier-to-autointegration factor isoform X1 [Equus caballus]	3

1333677258	collagen alpha-3(VI) chain isoform X1 [Equus caballus]	54	1333594974	basement membrane-specific heparan sulfate proteoglycan core protein isoform X6 [Equus caballus]	106
1333703017	collagen and calcium-binding EGF domain-containing protein 1 isoform X1 [Equus caballus]	2	28315888	beta-2-microglobulin [Equus caballus]	7
338718912	complement C1q tumor necrosis factor-related protein 3 isoform X1 [Equus caballus]	3	1333674519	bifunctional purine biosynthesis protein PURH [Equus caballus]	3
545219168	complement C1r subcomponent [Equus caballus]	19	1333596460	bone morphogenetic protein 1 isoform X1 [Equus caballus]	16
1333680053	complement C1s subcomponent [Equus caballus]	12	1333585664	BTB/POZ domain-containing protein KCTD12 [Equus caballus]	8
149732359	complement C4-A [Equus caballus]	7	1333560984	C-type mannose receptor 2 isoform X1 [Equus caballus]	3
149732066	complement factor B [Equus caballus]	38	1333702207	cadherin-2 [Equus caballus]	10
217454358	CXCL3 [Equus caballus]	2	953851391	caldesmon isoform X1 [Equus caballus]	5
1333604860	cystatin-C [Equus caballus]	7	1333613646	calmodulin-1 [Equus caballus]	5
1333567008	cystatin-M [Equus caballus]	2	149756873	calreticulin [Equus caballus]	10
149743847	cysteine and glycine-rich protein 1 [Equus caballus]	2	1333595729	calsyntenin-1 isoform X2 [Equus caballus]	11
1333603022	cytokine receptor-like factor 1 isoform X2 [Equus caballus]	13	149705852	calumenin isoform X1 [Equus caballus]	6
545215451	dermatopontin [Equus caballus]	5	338724227	calumenin isoform X2 [Equus caballus]	6
1333702213	desmocollin-2 isoform X1 [Equus caballus]	4	1333565326	catalase isoform X1 [Equus caballus]	3

1333698026	dickkopf-related protein 3 [Equus caballus]	4	545209689	cathepsin B isoform X1 [Equus caballus]	13
1333573738	dihydropyrimidinase-related protein 3 isoform X1 [Equus caballus]	4	1333568074	cathepsin D [Equus caballus]	2
1333581924	dystroglycan [Equus caballus]	4	1333609553	cathepsin L1 isoform X2 [Equus caballus]	17
1333579802	EGF-containing fibulin-like extracellular matrix protein 1 [Equus caballus]	13	149724398	caveolae-associated protein 1 [Equus caballus]	3
1333566953	EGF-containing fibulin-like extracellular matrix protein 2 [Equus caballus]	11	149731345	CD166 antigen isoform X1 [Equus caballus]	12
1333575331	EGF-like repeat and discoidin I-like domain-containing protein 3 isoform X1 [Equus caballus]	5	1333565145	CD44 antigen isoform X1 [Equus caballus]	2
1333706280	elongation factor 1-delta isoform X4 [Equus caballus]	2	338714429	cell growth regulator with EF hand domain protein 1 [Equus caballus]	4
1333690943	elongation factor 2 [Equus caballus]	4	338714528	cellular nucleic acid-binding protein isoform X2 [Equus caballus]	4
1333578946	EMILIN-1 [Equus caballus]	22	1279526421	Chain A, Serum albumin	34
255522883	endoplasmin precursor [Equus caballus]	3	260656201	Chain B, Gelsolin	6
1333567040	endosialin [Equus caballus]	6	545195240	chondroitin sulfate proteoglycan 4 [Equus caballus]	5
545200312	endothelial protein C receptor [Equus caballus]	5	545229460	chordin-like protein 1 isoform X1 [Equus caballus]	9
388260739	enolase 1 [Equus caballus]	16	1333655600	chromobox protein homolog 3 isoform X1 [Equus caballus]	3
1333613005	epididymal secretory protein E1 [Equus caballus]	6	1358101319	clathrin light chain A [Equus caballus]	2

1333666434	extracellular matrix protein 1 isoform X1 [Equus caballus]	13	1048721	clusterin [Equus caballus]	23
1333704580	extracellular sulfatase Sulf-1 isoform X1 [Equus caballus]	4	149725588	cofilin-1 [Equus caballus]	9
1333638625	extracellular superoxide dismutase [Cu-Zn] [Equus caballus]	3	1333552835	cofilin-2 isoform X1 [Equus caballus]	6
1333551284	fibrillin-1 isoform X1 [Equus caballus]	75	545192605	coiled-coil domain-containing protein 80 [Equus caballus]	3
1333574523	fibrillin-2 isoform X1 [Equus caballus]	5	1333562231	collagen alpha-1(I) chain [Equus caballus]	113
1333674527	fibronectin isoform X1 [Equus caballus]	99	643431288	collagen alpha-1(I), partial [Equus caballus]	4
1333626677	fibulin-1 isoform X1 [Equus caballus]	14	1333586033	collagen alpha-1(IV) chain [Equus caballus]	4
1333626681	fibulin-1 isoform X2 [Equus caballus]	13	1333617313	collagen alpha-1(V) chain isoform X1 [Equus caballus]	29
1333580472	fibulin-2 isoform X1 [Equus caballus]	15	1333617315	collagen alpha-1(V) chain isoform X2 [Equus caballus]	28
1333708655	filamin-A isoform X1 [Equus caballus]	15	194226345	collagen alpha-1(VI) chain [Equus caballus]	33
1333581278	filamin-B isoform X1 [Equus caballus]	2	1333556707	collagen alpha-1(XII) chain isoform X1 [Equus caballus]	19
194222800	follistatin-related protein 1 [Equus caballus]	14	1333618603	collagen alpha-1(XV) chain isoform X2 [Equus caballus]	4
1333690251	follistatin-related protein 3 [Equus caballus]	2	1333620681	collagen alpha-1(XVIII) chain isoform X1 [Equus caballus]	10
1333570053	fructose-bisphosphate aldolase A isoform X1 [Equus caballus]	9	149705490	collagen alpha-2(I) chain [Equus caballus]	101

953889609	galectin-1 [Equus caballus]	5	1333586031	collagen alpha-2(IV) chain [Equus caballus]	4
149723277	galectin-3-binding protein [Equus caballus]	12	149730792	collagen alpha-2(V) chain [Equus caballus]	47
126352460	ganglioside GM2 activator precursor [Equus caballus]	2	1333620786	collagen alpha-2(VI) chain isoform X1 [Equus caballus]	26
1333615354	gelsolin isoform X1 [Equus caballus]	18	1333696054	collagen alpha-3(V) chain [Equus caballus]	2
1333542062	glia-derived nexin isoform X1 [Equus caballus]	12	1333677258	collagen alpha-3(VI) chain isoform X1 [Equus caballus]	30
1333553844	glucose-6-phosphate isomerase [Equus caballus]	2	338718912	complement C1q tumor necrosis factor-related protein 3 isoform X1 [Equus caballus]	4
953854382	glutathione S-transferase Mu 1 isoform X1 [Equus caballus]	2	1333563850	complement component 1 Q subcomponent-binding protein, mitochondrial [Equus caballus]	2
255522848	glyceraldehyde-3-phosphate dehydrogenase [Equus caballus]	4	149732066	complement factor B [Equus caballus]	3
149723703	granulins isoform X1 [Equus caballus]	7	1333573454	copper transport protein ATOX1 [Equus caballus]	3
124377696	heat shock 70kDa protein 8 [Equus caballus]	12	1333604860	cystatin-C [Equus caballus]	5
149755998	heat shock protein beta-1 [Equus caballus]	2	1333567008	cystatin-M [Equus caballus]	4
1333611640	heat shock protein HSP 90-alpha isoform X1 [Equus caballus]	2	149743847	cysteine and glycine-rich protein 1 [Equus caballus]	4
1333613981	HHIP-like protein 1 isoform X1 [Equus caballus]	2	1333614987	cysteine-rich protein 1 [Equus caballus]	2

349603351	Histidine triad nucleotide-binding protein 1-like protein [Equus caballus]	2	1333614965	cysteine-rich protein 2 [Equus caballus]	2
1333598730	histone H2A type 1 [Equus caballus]	2	149721868	cytochrome c oxidase subunit 6B1 [Equus caballus]	2
545217098	histone H2B type 2-F [Equus caballus]	2	1333603020	cytokine receptor-like factor 1 isoform X1 [Equus caballus]	13
1333598753	histone H3.1 [Equus caballus]	4	822606514	cytosol aminopeptidase [Equus caballus]	2
1333601411	inactive tyrosine-protein kinase 7 [Equus caballus]	4	1333699825	D-dopachrome decarboxylase [Equus caballus]	2
508704	insulin-like growth factor II precursor, partial [Equus caballus]	2	1333545445	deleted in malignant brain tumors 1 protein-like [Equus caballus]	2
1333674713	insulin-like growth factor-binding protein 2 [Equus caballus]	15	545215451	dermatopontin [Equus caballus]	5
1333652689	insulin-like growth factor-binding protein 3 [Equus caballus]	8	1333702213	desmocollin-2 isoform X1 [Equus caballus]	2
1333561763	insulin-like growth factor-binding protein 4 [Equus caballus]	7	1333605125	destrin isoform X1 [Equus caballus]	2
820948134	insulin-like growth factor-binding protein 5 precursor [Equus caballus]	2	1333698026	dickkopf-related protein 3 [Equus caballus]	6
820948145	insulin-like growth factor-binding protein 6 precursor [Equus caballus]	12	149746221	dihydropyrimidinase-related protein 2 isoform X1 [Equus caballus]	10
149730228	integral membrane protein 2B [Equus caballus]	4	1333573738	dihydropyrimidinase-related protein 3 isoform X1 [Equus caballus]	13
338715409	integrin beta-like protein 1 [Equus caballus]	3	1333572754	drebrin isoform X1 [Equus caballus]	2
1333688737	L-lactate dehydrogenase A chain isoform X1 [Equus caballus]	2	1333581924	dystroglycan [Equus caballus]	5

1333549072	lactadherin isoform X2 [Equus caballus]	5	1333579802	EGF-containing fibulin-like extracellular matrix protein 1 [Equus caballus]	15
255653068	lactotransferrin precursor [Equus caballus]	2	1333566953	EGF-containing fibulin-like extracellular matrix protein 2 [Equus caballus]	12
149751386	lamin [Equus caballus]	5	1333575331	EGF-like repeat and discoidin I-like domain-containing protein 3 isoform X1 [Equus caballus]	3
545177549	laminin subunit alpha-2 isoform X3 [Equus caballus]	11	126352304	elongation factor 1-alpha 1 [Equus caballus]	4
1333557540	laminin subunit alpha-4 isoform X1 [Equus caballus]	32	1333587781	elongation factor 1-beta [Equus caballus]	3
1333651666	laminin subunit beta-1 [Equus caballus]	41	1333706280	elongation factor 1-delta isoform X4 [Equus caballus]	5
1333662823	laminin subunit gamma-1 [Equus caballus]	42	126352468	elongation factor 1-gamma [Equus caballus]	4
1333578628	latent-transforming growth factor beta-binding protein 1 isoform X1 [Equus caballus]	16	1333690943	elongation factor 2 [Equus caballus]	11
1333612986	latent-transforming growth factor beta-binding protein 2 isoform X1 [Equus caballus]	23	1333578946	EMILIN-1 [Equus caballus]	15
1333554469	latent-transforming growth factor beta-binding protein 4 isoform X1 [Equus caballus]	5	1333703666	endoplasmic reticulum resident protein 29 isoform X1 [Equus caballus]	4
1333600334	LOW QUALITY PROTEIN: heat shock 70 kDa protein 1-like [Equus caballus]	2	1333618655	endoplasmic reticulum resident protein 44 isoform X2 [Equus caballus]	2

1333695800	low-density lipoprotein receptor [Equus caballus]	6	255522883	endoplasmin precursor [Equus caballus]	12
124377698	lumican [Equus caballus]	22	1333567040	endosialin [Equus caballus]	7
1333550208	lysyl oxidase homolog 1 isoform X1 [Equus caballus]	9	545200312	endothelial protein C receptor [Equus caballus]	5
1333596550	lysyl oxidase homolog 2 isoform X1 [Equus caballus]	10	388260739	enolase 1 [Equus caballus]	28
193248596	M2-type pyruvate kinase [Equus caballus]	13	1333613005	epididymal secretory protein E1 [Equus caballus]	8
1333667727	macrophage colony-stimulating factor 1 isoform X1 [Equus caballus]	11	1333564220	eukaryotic initiation factor 4A-I isoform X1 [Equus caballus]	3
1333577320	macrophage-capping protein isoform X1 [Equus caballus]	2	1333564169	eukaryotic translation initiation factor 5A-1 [Equus caballus]	5
149713779	matrix Gla protein [Equus caballus]	2	149733215	eukaryotic translation initiation factor 6 isoform X1 [Equus caballus]	2
1333596256	matrix remodeling-associated protein 8 [Equus caballus]	8	1333666434	extracellular matrix protein 1 isoform X1 [Equus caballus]	14
1333709666	matrix-remodeling-associated protein 5 isoform X1 [Equus caballus]	48	1333638625	extracellular superoxide dismutase [Cu-Zn] [Equus caballus]	3
308912538	mesencephalic astrocyte-derived neurotrophic factor precursor [Equus caballus]	2	1333647971	ezrin [Equus caballus]	5
126722914	metalloproteinase inhibitor 1 precursor [Equus caballus]	11	1333667446	F-actin-capping protein subunit alpha-1 [Equus caballus]	2
1333559961	metalloproteinase inhibitor 2 [Equus caballus]	9	545208709	F-actin-capping protein subunit beta isoform X1 [Equus caballus]	2

1333559454	meteorin-like protein [Equus caballus]	3	1333670131	far upstream element-binding protein 1 isoform X1 [Equus caballus]	3
261490217	MHC class I antigen, partial [Equus caballus]	2	1333568827	fascin [Equus caballus]	11
1333595292	microfibrillar-associated protein 2 isoform X1 [Equus caballus]	2	149721423	fatty acid-binding protein, epidermal [Equus caballus]	6
149755658	moesin [Equus caballus]	6	1333551284	fibrillin-1 isoform X1 [Equus caballus]	86
1333573985	monocyte differentiation antigen CD14-like [Equus caballus]	5	1333574523	fibrillin-2 isoform X1 [Equus caballus]	11
545193779	multiple inositol polyphosphate phosphatase 1 isoform X1 [Equus caballus]	2	255522903	fibroblast growth factor 7 [Equus caballus]	2
1333624162	myosin-9 [Equus caballus]	8	1333674527	fibronectin isoform X1 [Equus caballus]	110
1333550247	neogenin isoform X1 [Equus caballus]	2	1333626677	fibulin-1 isoform X1 [Equus caballus]	16
1333566330	neuroblast differentiation-associated protein AHNAK isoform X3 [Equus caballus]	5	1333626681	fibulin-1 isoform X2 [Equus caballus]	16
338712486	neutral alpha-glucosidase AB isoform X1 [Equus caballus]	2	1333580472	fibulin-2 isoform X1 [Equus caballus]	18
1333548199	nidogen-1 [Equus caballus]	19	1333708655	filamin-A isoform X1 [Equus caballus]	49
1333553314	nidogen-2 isoform X1 [Equus caballus]	11	1333581278	filamin-B isoform X1 [Equus caballus]	8
545192207	nmrA-like family domain-containing protein 1 [Equus caballus]	4	1333657250	filamin-C isoform X1 [Equus caballus]	6

1333555293	nucleobindin-1 [Equus caballus]	14	338710456	flavin reductase (NADPH) [Equus caballus]	2
335775016	nucleobindin-2-like protein [Equus caballus]	4	194222800	follistatin-related protein 1 [Equus caballus]	18
1333559399	nucleoside diphosphate kinase B isoform X1 [Equus caballus]	2	1333690251	follistatin-related protein 3 [Equus caballus]	4
545217394	olfactomedin-like protein 3 isoform X1 [Equus caballus]	17	1333570053	fructose-bisphosphate aldolase A isoform X1 [Equus caballus]	13
1333694060	out at first protein homolog [Equus caballus]	8	1333548923	furin [Equus caballus]	2
1333589748	pentraxin-related protein PTX3 [Equus caballus]	7	953889609	galectin-1 [Equus caballus]	10
953850207	peptidyl-prolyl cis-trans isomerase A [Equus caballus]	8	149723277	galectin-3-binding protein [Equus caballus]	3
194216907	peptidyl-prolyl cis-trans isomerase FKBP10 [Equus caballus]	9	126352460	ganglioside GM2 activator precursor [Equus caballus]	2
148529811	peptidylprolyl isomerase B [Equus caballus]	5	1333615354	gelsolin isoform X1 [Equus caballus]	19
1333579729	peroxidasin homolog isoform X1 [Equus caballus]	35	1333542062	glia-derived nexin isoform X1 [Equus caballus]	5
149693696	peroxiredoxin-1 [Equus caballus]	5	1333553844	glucose-6-phosphate isomerase [Equus caballus]	4
1333695489	peroxiredoxin-2 isoform X1 [Equus caballus]	2	545222182	glucosidase 2 subunit beta isoform X1 [Equus caballus]	3
1333707197	peroxiredoxin-4 isoform X1 [Equus caballus]	3	1333548627	glutamate dehydrogenase 1, mitochondrial [Equus caballus]	3
149707887	peroxiredoxin-6 [Equus caballus]	2	1333622075	glutathione reductase, mitochondrial isoform X1 [Equus caballus]	2

149720563	phosphatidylethanolamine-binding protein 1 [Equus caballus]	2	149708716	glutathione S-transferase Mu 1 [Equus caballus]	2
1333709621	phosphoglycerate kinase 1 [Equus caballus]	7	255522848	glyceraldehyde-3-phosphate dehydrogenase [Equus caballus]	14
83744154	pigment epithelium-derived factor [Equus caballus]	18	1333656071	glycine--tRNA ligase [Equus caballus]	2
149758084	plasma protease C1 inhibitor [Equus caballus]	8	225543240	glycogen phosphorylase, liver form [Equus caballus]	3
1333569276	plasminogen activator inhibitor 1 [Equus caballus]	13	1333677851	glypican-1 [Equus caballus]	4
338728078	plasminogen activator inhibitor 2 [Equus caballus]	5	149723703	granulins isoform X1 [Equus caballus]	6
1333563619	platelet-activating factor acetylhydrolase IB subunit alpha [Equus caballus]	2	545210415	gremlin-2 [Equus caballus]	2
1333706431	plectin isoform X5 [Equus caballus]	5	1333701536	GTP-binding nuclear protein Ran [Equus caballus]	2
1333544015	pro-cathepsin H [Equus caballus]	3	149699777	haptoglobin [Equus caballus]	2
1333569206	procollagen C-endopeptidase enhancer 1 isoform X1 [Equus caballus]	24	124377696	heat shock 70kDa protein 8 [Equus caballus]	24
545209074	procollagen-lysine,2-oxoglutarate 5-dioxygenase 1 isoform X1 [Equus caballus]	14	37142918	heat shock protein 90 [Equus caballus]	4
1333569285	procollagen-lysine,2-oxoglutarate 5-dioxygenase 3 isoform X2 [Equus caballus]	22	149755998	heat shock protein beta-1 [Equus caballus]	8
953868285	profilin-1 [Equus caballus]	3	1333611640	heat shock protein HSP 90-alpha isoform X1 [Equus caballus]	4
545215255	prolargin [Equus caballus]	2	1333664682	hepatoma-derived growth factor [Equus caballus]	2

1333686460	prolow-density lipoprotein receptor-related protein 1 isoform X1 [Equus caballus]	11	1333572687	heterogeneous nuclear ribonucleoprotein A/B isoform X1 [Equus caballus]	4
1333547563	prolyl 4-hydroxylase subunit alpha-1 isoform X1 [Equus caballus]	2	953856128	heterogeneous nuclear ribonucleoprotein A1 isoform X1 [Equus caballus]	4
953884791	proprotein convertase subtilisin/kexin type 5 isoform X1 [Equus caballus]	3	1333636282	heterogeneous nuclear ribonucleoprotein D-like isoform X1 [Equus caballus]	3
1333707828	proSAAS [Equus caballus]	3	1333636298	heterogeneous nuclear ribonucleoprotein D0 isoform X1 [Equus caballus]	5
545193979	prosaposin isoform X3 [Equus caballus]	5	1333572600	heterogeneous nuclear ribonucleoprotein H isoform X1 [Equus caballus]	3
149756583	protein canopy homolog 2 [Equus caballus]	2	1333609317	heterogeneous nuclear ribonucleoprotein K isoform X1 [Equus caballus]	3
194211151	protein CYR61 [Equus caballus]	3	1333556986	heterogeneous nuclear ribonucleoprotein Q isoform X1 [Equus caballus]	2
1333559663	protein disulfide-isomerase [Equus caballus]	12	1333644394	heterogeneous nuclear ribonucleoprotein U isoform X1 [Equus caballus]	4
1333551507	protein disulfide-isomerase A3 [Equus caballus]	7	1333566391	heterogeneous nuclear ribonucleoprotein U-like protein 2 isoform X1 [Equus caballus]	2
1333579492	protein disulfide-isomerase A6 [Equus caballus]	2	1333655583	heterogeneous nuclear ribonucleoproteins A2/B1 isoform X1 [Equus caballus]	8

149751468	protein S100-A11 [Equus caballus]	4	1333613981	HHIP-like protein 1 isoform X1 [Equus caballus]	2
545216843	protein S100-A4 [Equus caballus]	2	335775462	high mobility group protein B1-like protein [Equus caballus]	5
1333574702	protein-lysine 6-oxidase isoform X1 [Equus caballus]	4	1333600039	histone H1.3 [Equus caballus]	2
1333595794	protein/nucleic acid deglycase DJ-1 isoform X1 [Equus caballus]	2	149754742	histone H1.5 [Equus caballus]	2
1333663018	proteoglycan 4 [Equus caballus]	5	1333598730	histone H2A type 1 [Equus caballus]	8
1333630825	rab GDP dissociation inhibitor beta isoform X2 [Equus caballus]	2	545197429	histone H2A type 1-E [Equus caballus]	4
38604884	RecName: Full=Annexin A1; AltName: Full=Annexin I; AltName: Full=Annexin-1; AltName: Full=Calpactin II; AltName: Full=Calpactin-2; AltName: Full=Lipocortin I	13	1333635392	histone H2A.Z [Equus caballus]	2
8134615	RecName: Full=Biglycan; AltName: Full=Bone/cartilage proteoglycan I; AltName: Full=PG-S1; Flags: Precursor	26	1333693950	histone H2AX [Equus caballus]	4
6093705	RecName: Full=Decorin; AltName: Full=Bone proteoglycan II; AltName: Full=Dermatan sulfate proteoglycan II; Short=DS-PGII; AltName: Full=PG-S2; Flags: Precursor	18	1333598737	histone H2B type 1-J [Equus caballus]	3
124616	RecName: Full=Insulin; Contains: RecName: Full=Insulin B chain; Contains: RecName: Full=Insulin A chain; Flags: Precursor	2	1333598753	histone H3.1 [Equus caballus]	10

110810385	RecName: Full=Transferrin receptor protein 1; Short=TR; Short=TfR; Short=TfR1; Short=Trfr; AltName: CD_antigen=CD71	2	1333599946	histone H3.1 [Equus caballus]	2
1333698495	reticulocalbin-1 isoform X1 [Equus caballus]	3	1333687607	histone H3.3 [Equus caballus]	2
1333555461	reticulocalbin-3 [Equus caballus]	2	335775311	histone-binding protein RBBP4-like protein [Equus caballus]	2
1333542798	retinoic acid receptor responder protein 1 [Equus caballus]	4	823270915	hsc70-interacting protein [Equus caballus]	2
1333542792	retinoic acid receptor responder protein 1-like isoform X1 [Equus caballus]	3	545221374	hypoxia up-regulated protein 1 isoform X1 [Equus caballus]	3
1333658843	retinoic acid receptor responder protein 2 [Equus caballus]	5	1333601411	inactive tyrosine-protein kinase 7 [Equus caballus]	10
1333546841	retinol-binding protein 4 isoform X1 [Equus caballus]	3	126352436	inhibin beta A chain precursor [Equus caballus]	2
1333559671	rho GDP-dissociation inhibitor 1 [Equus caballus]	3	1333567577	insulin [Equus caballus]	3
545194374	ribonuclease 4 [Equus caballus]	5	56849564	insulin-like growth factor binding protein-4, partial [Equus caballus]	5
1333648020	ribonuclease T2 [Equus caballus]	2	1333674713	insulin-like growth factor-binding protein 2 [Equus caballus]	21
1333650985	semaphorin-3C isoform X1 [Equus caballus]	10	1333652689	insulin-like growth factor-binding protein 3 [Equus caballus]	8
194206477	semaphorin-7A [Equus caballus]	3	1333561763	insulin-like growth factor-binding protein 4 [Equus caballus]	9
544066294	Sequence 14 from patent US 8501157	14	820948134	insulin-like growth factor-binding protein 5 precursor [Equus caballus]	2

1333545455	serine protease HTRA1 [Equus caballus]	19	820948145	insulin-like growth factor-binding protein 6 precursor [Equus caballus]	11
1333599335	serpin B6 [Equus caballus]	2	149730228	integral membrane protein 2B [Equus caballus]	2
1333697122	serpin H1 [Equus caballus]	18	1333685413	integrin alpha-5 [Equus caballus]	2
126723746	serum albumin precursor [Oryctolagus cuniculus]	12	338715409	integrin beta-like protein 1 [Equus caballus]	4
1333698300	serum amyloid A protein isoform X1 [Equus caballus]	3	545190768	isocitrate dehydrogenase [NADP] cytoplasmic isoform X1 [Equus caballus]	3
255522921	serum amyloid A protein precursor [Equus caballus]	3	1333688737	L-lactate dehydrogenase A chain isoform X1 [Equus caballus]	3
149719493	serum amyloid A protein-like [Equus caballus]	2	255653068	lactotransferrin precursor [Equus caballus]	3
99109459	SPARC [Equus caballus]	20	149751386	lamin [Equus caballus]	20
1333698107	spondin-1 isoform X1 [Equus caballus]	2	1333574578	lamin-B1 [Equus caballus]	3
1333640297	spondin-2 [Equus caballus]	5	545177549	laminin subunit alpha-2 isoform X3 [Equus caballus]	18
1333573027	stanniocalcin-2 [Equus caballus]	6	1333557540	laminin subunit alpha-4 isoform X1 [Equus caballus]	23
1333662591	sulfhydryl oxidase 1 [Equus caballus]	13	1333651666	laminin subunit beta-1 [Equus caballus]	39
126352669	superoxide dismutase [Cu-Zn] [Equus caballus]	2	1333581973	laminin subunit beta-2 isoform X1 [Equus caballus]	2
1333710533	sushi repeat-containing protein SRPX isoform X1 [Equus caballus]	5	1333662823	laminin subunit gamma-1 [Equus caballus]	42
1333709656	sushi repeat-containing protein SRPX2 [Equus caballus]	5	1333578628	latent-transforming growth factor beta-binding protein 1 isoform X1 [Equus caballus]	13

1333561518	synaptic vesicle membrane protein VAT-1 homolog [Equus caballus]	2	1333612986	latent-transforming growth factor beta-binding protein 2 isoform X1 [Equus caballus]	22
1333704040	syntenin-1 isoform X1 [Equus caballus]	3	1333566893	latent-transforming growth factor beta-binding protein 3 isoform X1 [Equus caballus]	2
1333591700	target of Nesh-SH3 isoform X36 [Equus caballus]	7	1333554469	latent-transforming growth factor beta-binding protein 4 isoform X1 [Equus caballus]	6
1333615758	tenascin isoform X1 [Equus caballus]	33	1333561983	LIM and SH3 domain protein 1 isoform X1 [Equus caballus]	3
126352340	thioredoxin [Equus caballus]	3	1333683921	LIM domain and actin-binding protein 1 isoform X1 [Equus caballus]	3
1333599540	thioredoxin domain-containing protein 5 [Equus caballus]	2	1333589629	lipoma-preferred partner [Equus caballus]	4
822092742	thrombospondin-1 precursor [Equus caballus]	16	1333600334	LOW QUALITY PROTEIN: heat shock 70 kDa protein 1-like [Equus caballus]	5
824556506	thrombospondin-2 precursor [Equus caballus]	24	1333666625	LOW QUALITY PROTEIN: histone H3 [Equus caballus]	2
1333665097	thrombospondin-3 isoform X1 [Equus caballus]	2	1333614134	LOW QUALITY PROTEIN: protein AHNAK2 [Equus caballus]	5
1333694044	thy-1 membrane glycoprotein [Equus caballus]	2	1333695800	low-density lipoprotein receptor [Equus caballus]	9
149705478	tissue factor pathway inhibitor 2 [Equus caballus]	2	124377698	lumican [Equus caballus]	19
6176280	transferrin, partial [Equus caballus]	3	1333550208	lysyl oxidase homolog 1 isoform X1 [Equus caballus]	14

1333669017	transforming growth factor beta receptor type 3 isoform X1 [Equus caballus]	2	1333596550	lysyl oxidase homolog 2 isoform X1 [Equus caballus]	16
1333574238	transforming growth factor-beta-induced protein ig-h3 isoform X2 [Equus caballus]	26	545186364	lysyl oxidase homolog 3 isoform X1 [Equus caballus]	4
158328502	transgelin [Equus caballus]	7	193248596	M2-type pyruvate kinase [Equus caballus]	32
953853468	transgelin-2 [Equus caballus]	6	1333667727	macrophage colony-stimulating factor 1 isoform X1 [Equus caballus]	9
1333618130	transitional endoplasmic reticulum ATPase [Equus caballus]	4	149720186	macrophage migration inhibitory factor [Equus caballus]	2
1333581483	transketolase [Equus caballus]	3	1333577320	macrophage-capping protein isoform X1 [Equus caballus]	4
194211629	triosephosphate isomerase [Equus caballus]	11	149727540	malate dehydrogenase, cytoplasmic [Equus caballus]	2
1333550786	tropomyosin alpha-1 chain isoform X1 [Equus caballus]	5	306922412	malate dehydrogenase, mitochondrial [Equus caballus]	8
149751316	tropomyosin alpha-3 chain isoform X10 [Equus caballus]	6	149713779	matrix Gla protein [Equus caballus]	2
1333602522	tropomyosin alpha-4 chain isoform X2 [Equus caballus]	10	1333596256	matrix remodeling-associated protein 8 [Equus caballus]	12
149739249	tropomyosin beta chain isoform X4 [Equus caballus]	7	1333709666	matrix-remodeling-associated protein 5 isoform X1 [Equus caballus]	39
953859478	tsukushin isoform X1 [Equus caballus]	2	308912538	mesencephalic astrocyte-derived neurotrophic factor precursor [Equus caballus]	3
149694136	tubulointerstitial nephritis antigen-like [Equus caballus]	2	126722914	metalloproteinase inhibitor 1 precursor [Equus caballus]	14

62856981	tumor necrosis factor alpha-induced protein 6 [Equus caballus]	7	1333559961	metalloproteinase inhibitor 2 [Equus caballus]	8
15408577	type I collagen alpha 2 chain, partial [Equus caballus]	11	1333559454	meteorin-like protein [Equus caballus]	4
545175634	tyrosine-protein kinase receptor UFO isoform X1 [Equus caballus]	4	1333595292	microfibrillar-associated protein 2 isoform X1 [Equus caballus]	2
338716897	urokinase-type plasminogen activator [Equus caballus]	15	1333575805	microtubule-associated protein 1B isoform X1 [Equus caballus]	6
1333707051	V-type proton ATPase subunit S1 [Equus caballus]	3	1333582151	microtubule-associated protein 4 isoform X1 [Equus caballus]	3
154756883	vascular cell adhesion molecule [Equus caballus]	3	149755658	moesin [Equus caballus]	10
1333598384	vascular endothelial growth factor A isoform X1 [Equus caballus]	2	1333691247	myeloid-derived growth factor [Equus caballus]	2
1333621957	vascular endothelial growth factor C isoform X1 [Equus caballus]	4	149756573	myosin light polypeptide 6 isoform X2 [Equus caballus]	5
1333571101	vasorin [Equus caballus]	6	953861232	myosin regulatory light polypeptide 9 isoform X1 [Equus caballus]	3
1333575337	versican core protein isoform X1 [Equus caballus]	25	1333624162	myosin-9 [Equus caballus]	35
1333610324	very low-density lipoprotein receptor isoform X1 [Equus caballus]	3	149747438	myotrophin [Equus caballus]	2
149726043	vesicular integral-membrane protein VIP36 isoform X1 [Equus caballus]	2	1333550247	neogenin isoform X1 [Equus caballus]	2
341865569	vimentin [Equus caballus]	23	1333664708	nestin [Equus caballus]	2
1333544627	vinculin isoform X1 [Equus caballus]	8	953857785	neural cell adhesion molecule 1 isoform X1 [Equus caballus]	2

1333639049	WD repeat-containing protein 1 [Equus caballus]	2
1333607375	WNT1-inducible-signaling pathway protein 2 [Equus caballus]	2
1333566330	neuroblast differentiation-associated protein AHNAK isoform X3 [Equus caballus]	31
194218317	neutral alpha-glucosidase AB isoform X2 [Equus caballus]	6
1333548199	nidogen-1 [Equus caballus]	23
1333553314	nidogen-2 isoform X1 [Equus caballus]	5
545192207	nmrA-like family domain-containing protein 1 [Equus caballus]	5
1333593091	nuclear autoantigenic sperm protein isoform X1 [Equus caballus]	2
1333593438	nuclease-sensitive element-binding protein 1 isoform X1 [Equus caballus]	4
1333555293	nucleobindin-1 [Equus caballus]	15
1333688719	nucleobindin-2 isoform X1 [Equus caballus]	2
1333676639	nucleolin [Equus caballus]	4
953872575	nucleophosmin isoform X1 [Equus caballus]	2
545177882	nucleoside diphosphate kinase A isoform X1 [Equus caballus]	2
1333559399	nucleoside diphosphate kinase B isoform X1 [Equus caballus]	3
545217394	olfactomedin-like protein 3 isoform X1 [Equus caballus]	16

1333694060	out at first protein homolog [Equus caballus]	2
1333635619	PDZ and LIM domain protein 5 isoform X1 [Equus caballus]	2
1333572775	PDZ and LIM domain protein 7 isoform X1 [Equus caballus]	2
953850207	peptidyl-prolyl cis-trans isomerase A [Equus caballus]	9
194216907	peptidyl-prolyl cis-trans isomerase FKBP10 [Equus caballus]	9
1333605756	peptidyl-prolyl cis-trans isomerase FKBP1A isoform X2 [Equus caballus]	5
545181560	peptidyl-prolyl cis-trans isomerase FKBP2 isoform X1 [Equus caballus]	2
1333553095	peptidyl-prolyl cis-trans isomerase FKBP3 isoform X1 [Equus caballus]	2
1333586748	peptidyl-prolyl cis-trans isomerase FKBP7 isoform X2 [Equus caballus]	3
148529811	peptidylprolyl isomerase B [Equus caballus]	8
1333579729	peroxidasin homolog isoform X1 [Equus caballus]	23
149693696	peroxiredoxin-1 [Equus caballus]	5
1333707197	peroxiredoxin-4 isoform X1 [Equus caballus]	5

149707887	peroxiredoxin-6 [Equus caballus]	3
149720563	phosphatidylethanolamine-binding protein 1 [Equus caballus]	10
1333546556	phosphoglycerate mutase 1 [Equus caballus]	7
83744154	pigment epithelium-derived factor [Equus caballus]	18
1333569276	plasminogen activator inhibitor 1 [Equus caballus]	12
338728078	plasminogen activator inhibitor 2 [Equus caballus]	5
149744913	plastin-3 [Equus caballus]	3
1333563619	platelet-activating factor acetylhydrolase IB subunit alpha [Equus caballus]	4
1333706431	plectin isoform X5 [Equus caballus]	36
1333705365	polyadenylate-binding protein 1 [Equus caballus]	6
149759819	prefoldin subunit 2 [Equus caballus]	2
1333544015	pro-cathepsin H [Equus caballus]	2
2653643	procollagen alpha 1 (I), partial [Equus caballus]	12
1333569206	procollagen C-endopeptidase enhancer 1 isoform X1 [Equus caballus]	26
545209074	procollagen-lysine,2-oxoglutarate 5-dioxygenase 1 isoform X1 [Equus caballus]	8

1333583841	procollagen-lysine,2-oxoglutarate 5-dioxygenase 2 isoform X1 [Equus caballus]	2
1333569285	procollagen-lysine,2-oxoglutarate 5-dioxygenase 3 isoform X2 [Equus caballus]	15
953868285	profilin-1 [Equus caballus]	8
1333553761	programmed cell death protein 5 [Equus caballus]	2
1333686460	prolow-density lipoprotein receptor-related protein 1 isoform X1 [Equus caballus]	11
1333547563	prolyl 4-hydroxylase subunit alpha-1 isoform X1 [Equus caballus]	9
1333574446	prolyl 4-hydroxylase subunit alpha-2 isoform X5 [Equus caballus]	5
1333707828	proSAAS [Equus caballus]	7
149689950	prosaposin isoform X1 [Equus caballus]	4
149692835	proteasome subunit alpha type-6 isoform X2 [Equus caballus]	3
149721035	proteasome subunit alpha type-7-like [Equus caballus]	3
149756583	protein canopy homolog 2 [Equus caballus]	5
1333583506	protein CDV3 homolog isoform X1 [Equus caballus]	2
194211151	protein CYR61 [Equus caballus]	5

1333559663	protein disulfide-isomerase [Equus caballus]	28
1333551507	protein disulfide-isomerase A3 [Equus caballus]	21
1333658626	protein disulfide-isomerase A4 [Equus caballus]	6
1333579492	protein disulfide-isomerase A6 [Equus caballus]	3
149751468	protein S100-A11 [Equus caballus]	4
545216843	protein S100-A4 [Equus caballus]	3
953852735	protein S100-A7 isoform X1 [Equus caballus]	2
149728927	protein shisa-5 isoform X1 [Equus caballus]	3
1333574702	protein-lysine 6-oxidase isoform X1 [Equus caballus]	7
149695427	protein/nucleic acid deglycase DJ-1 isoform X3 [Equus caballus]	6
1333707019	rab GDP dissociation inhibitor alpha [Equus caballus]	4
1333630825	rab GDP dissociation inhibitor beta isoform X2 [Equus caballus]	7
1333699580	ran-specific GTPase-activating protein isoform X1 [Equus caballus]	3
1333548900	ras GTPase-activating-like protein IQGAP1 isoform X1 [Equus caballus]	3

38604884	RecName: Full=Annexin A1; AltName: Full=Annexin I; AltName: Full=Annexin-1; AltName: Full=Calpactin II; AltName: Full=Calpactin-2; AltName: Full=Lipocortin I	26
8134615	RecName: Full=Biglycan; AltName: Full=Bone/cartilage proteoglycan I; AltName: Full=PG-S1; Flags: Precursor	26
6093705	RecName: Full=Decorin; AltName: Full=Bone proteoglycan II; AltName: Full=Dermatan sulfate proteoglycan II; Short=DS-PGII; AltName: Full=PG-S2; Flags: Precursor	19
124616	RecName: Full=Insulin; Contains: RecName: Full=Insulin B chain; Contains: RecName: Full=Insulin A chain; Flags: Precursor	3
52783777	RecName: Full=Phosphoglycerate kinase 1	16
85700159	RecName: Full=Thymosin beta-4; Short=T beta-4; Contains: RecName: Full=Hematopoietic system regulatory peptide; AltName: Full=Seraspenide	2
1333698495	reticulocalbin-1 isoform X1 [Equus caballus]	4
1333555461	reticulocalbin-3 [Equus caballus]	6
1333542798	retinoic acid receptor responder protein 1 [Equus caballus]	3

1333542792	retinoic acid receptor responder protein 1-like isoform X1 [Equus caballus]	3
1333658843	retinoic acid receptor responder protein 2 [Equus caballus]	8
1333546841	retinol-binding protein 4 isoform X1 [Equus caballus]	2
1333559671	rho GDP-dissociation inhibitor 1 [Equus caballus]	6
545194374	ribonuclease 4 [Equus caballus]	2
1333605121	ribosome-binding protein 1 isoform X1 [Equus caballus]	6
1333709971	RNA-binding motif protein, X chromosome [Equus caballus]	2
149744489	RNA-binding protein 3 [Equus caballus]	2
1333666081	selenium-binding protein 1 [Equus caballus]	3
953851051	semaphorin-3A isoform X1 [Equus caballus]	2
1333650985	semaphorin-3C isoform X1 [Equus caballus]	14
194206477	semaphorin-7A [Equus caballus]	5
544066294	Sequence 14 from patent US 8501157	17
1333545455	serine protease HTRA1 [Equus caballus]	15
1333599335	serpin B6 [Equus caballus]	6

1333697122	serpin H1 [Equus caballus]	21
149744781	SH3 domain-binding glutamic acid-rich-like protein isoform X1 [Equus caballus]	2
1333556437	soluble scavenger receptor cysteine-rich domain-containing protein SSC5D [Equus caballus]	8
99109459	SPARC [Equus caballus]	25
1333616606	spectrin alpha chain, non-erythrocytic 1 isoform X1 [Equus caballus]	3
1333578208	spectrin beta chain, non-erythrocytic 1 isoform X1 [Equus caballus]	3
1333566687	splicing factor 1 isoform X3 [Equus caballus]	2
1333640297	spondin-2 [Equus caballus]	5
255522905	stress-70 protein, mitochondrial [Equus caballus]	4
1333566532	stress-induced-phosphoprotein 1 [Equus caballus]	3
1333662591	sulfhydryl oxidase 1 [Equus caballus]	7
126352669	superoxide dismutase [Cu-Zn] [Equus caballus]	3
1333710533	sushi repeat-containing protein SRPX isoform X1 [Equus caballus]	6
1333709656	sushi repeat-containing protein SRPX2 [Equus caballus]	6

149743996	T-complex protein 1 subunit alpha isoform X1 [Equus caballus]	2
1333687568	T-complex protein 1 subunit beta [Equus caballus]	4
1333578015	T-complex protein 1 subunit delta [Equus caballus]	2
149751396	T-complex protein 1 subunit gamma [Equus caballus]	2
149742207	T-complex protein 1 subunit theta [Equus caballus]	2
1333618298	talin-1 isoform X1 [Equus caballus]	6
1333591700	target of Nesh-SH3 isoform X36 [Equus caballus]	6
1333615758	tenascin isoform X1 [Equus caballus]	28
126352340	thioredoxin [Equus caballus]	6
149724198	thioredoxin domain-containing protein 17 [Equus caballus]	3
1333599540	thioredoxin domain-containing protein 5 [Equus caballus]	8
212549578	thioredoxin reductase 1, cytoplasmic [Equus caballus]	2
822092742	thrombospondin-1 precursor [Equus caballus]	23
824556506	thrombospondin-2 precursor [Equus caballus]	28
1333665097	thrombospondin-3 isoform X1 [Equus caballus]	2

149705478	tissue factor pathway inhibitor 2 [Equus caballus]	4
1333574238	transforming growth factor-beta-induced protein ig-h3 isoform X2 [Equus caballus]	17
158328502	transgelin [Equus caballus]	9
953853468	transgelin-2 [Equus caballus]	10
1333618130	transitional endoplasmic reticulum ATPase [Equus caballus]	20
1333581483	transketolase [Equus caballus]	7
1333585361	translationally-controlled tumor protein isoform X1 [Equus caballus]	5
194211629	triosephosphate isomerase [Equus caballus]	21
1333550798	tropomyosin alpha-1 chain isoform X7 [Equus caballus]	14
149751316	tropomyosin alpha-3 chain isoform X10 [Equus caballus]	14
1333602522	tropomyosin alpha-4 chain isoform X2 [Equus caballus]	15
149739249	tropomyosin beta chain isoform X4 [Equus caballus]	11
338720079	tryptophan--tRNA ligase, cytoplasmic [Equus caballus]	8
149754673	tubulin beta chain [Equus caballus]	11
1333617779	tubulin beta-4B chain [Equus caballus]	9

1333575556	tubulin-specific chaperone A [Equus caballus]	2
149694136	tubulointerstitial nephritis antigen-like [Equus caballus]	6
62856981	tumor necrosis factor alpha-induced protein 6 [Equus caballus]	3
1333682825	twinfilin-1 [Equus caballus]	2
15408577	type I collagen alpha 2 chain, partial [Equus caballus]	12
545175634	tyrosine-protein kinase receptor UFO isoform X1 [Equus caballus]	6
1333709529	ubiquitin-like modifier-activating enzyme 1 [Equus caballus]	2
1333592914	UMP-CMP kinase [Equus caballus]	4
1333599948	uncharacterized protein LOC100054378 [Equus caballus]	2
310898507	unnamed protein product [Equus caballus]	2
338716897	urokinase-type plasminogen activator [Equus caballus]	18
1333543645	UV excision repair protein RAD23 homolog B [Equus caballus]	2
1333598384	vascular endothelial growth factor A isoform X1 [Equus caballus]	2
1333621957	vascular endothelial growth factor C isoform X1 [Equus caballus]	3
1333554876	vasodilator-stimulated phosphoprotein isoform X1 [Equus caballus]	2

1333571101	vasorin [Equus caballus]	5
1333575341	versican core protein isoform X3 [Equus caballus]	32
1333610324	very low-density lipoprotein receptor isoform X1 [Equus caballus]	5
149726043	vesicular integral-membrane protein VIP36 isoform X1 [Equus caballus]	3
341865569	vimentin [Equus caballus]	41
1333544627	vinculin isoform X1 [Equus caballus]	14
1333639049	WD repeat-containing protein 1 [Equus caballus]	15
1333607375	WNT1-inducible-signaling pathway protein 2 [Equus caballus]	3
1333680713	Y-box-binding protein 3 isoform X1 [Equus caballus]	2
